# Supplementary material for: Factors associated with changes in walking performance in individuals 3 months after stroke or TIA: secondary analyses from a randomised controlled trial of SMS-delivered training instructions in Sweden
Source: BMJ Open. 2024 Mar 4;14(3):e078180. doi: 10.1136/bmjopen-2023-078180 (PMC11146402; doi:10.1136/bmjopen-2023-078180)
Supplement: Author's manuscript [file bmjopen-2023-078180.draft_revisions.pdf]

BMJ Open is committed to open peer review. As part of this commitment we make the peer review history of every article we publish publicly available.

When an article is published we post the peer reviewers' comments and the authors' responses online. We also post the versions of the paper that were used during peer review. These are the versions that the peer review comments apply to.

The versions of the paper that follow are the versions that were submitted during the peer review process. They are not the versions of record or the final published versions. They should not be cited or distributed as the published version of this manuscript.

BMJ Open is an open access journal and the full, final, typeset and author-corrected version of record of the manuscript is available on our site with no access controls, subscription charges or pay-per-view fees (http://bmjopen.bmj.com).

If you have any questions on BMJ Open's open peer review process please email info.bmjopen@bmj.com

BMJ Open

Factors associated with changes in walking performance in individuals three months after stroke or TIA—secondary analyses from a randomized controlled trial of SMS-delivered training instructions

Journal:	*BMJ Open*
Manuscript ID	bmjopen-2023-078180
Article Type:	Original research
Date Submitted by the Author:	26-Jul-2023
Complete List of Authors:	Vahlberg, Birgit; Uppsala Universitet Medicinska fakulteten, Department of Public Health and Caring Sciences,
Keywords:	Stroke < NEUROLOGY, Risk Factors, REHABILITATION MEDICINE, Clinical Trial

SCHOLARONE™
Manuscripts

I, the Submitting Author has the right to grant and does grant on behalf of all authors of the Work (as defined in the below author licence), an exclusive licence and/or a non-exclusive licence for contributions from authors who are: i) UK Crown employees; ii) where BMJ has agreed a CC-BY licence shall apply, and/or iii) in accordance with the terms applicable for US Federal Government officers or employees acting as part of their official duties; on a worldwide, perpetual, irrevocable, royalty-free basis to BMJ Publishing Group Ltd ("BMJ") its licensees and where the relevant Journal is co-owned by BMJ to the co-owners of the Journal, to publish the Work in this journal and any other BMJ products and to exploit all rights, as set out in our licence.

The Submitting Author accepts and understands that any supply made under these terms is made by BMJ to the Submitting Author unless you are acting as an employee on behalf of your employer or a postgraduate student of an affiliated institution which is paying any applicable article publishing charge ("APC") for Open Access articles. Where the Submitting Author wishes to make the Work available on an Open Access basis (and intends to pay the relevant APC), the terms of reuse of such Open Access shall be governed by a Creative Commons licence – details of these licences and which Creative Commons licence will apply to this Work are set out in our licence referred to above.

Other than as permitted in any relevant BMJ Author's Self Archiving Policies, I confirm this Work has not been accepted for publication elsewhere, is not being considered for publication elsewhere and does not duplicate material already published. I confirm all authors consent to publication of this Work and authorise the granting of this licence.

For peer review only

Factors associated with changes in walking performance in individuals three months after stroke or TIA—secondary analyses from a randomized controlled trial of SMS-delivered training instructions

Short title: Predicting changes in walking performance three months after recent stroke and TIA

Corresponding author: Birgit Vahlberg

Address: Department of Public Health and Caring Sciences, Geriatrics,

Uppsala University, BMC A9

S-751 24 Uppsala, Sweden

Telephone: +46 709583473

E-mail: Birgit.Vahlberg@pubcare.uu.se

Word count main text: 2438~~xxxx~~

For peer review only

ABSTRACT:

Objectives: To identify factors related to changes in walking performance in individuals 3 months after a stroke or TIA.

Design: Cross-sectional study with post-hoc analysis of a randomized controlled study.

Setting: University Hospital, Sweden.

Participants: 79 individuals, 64 (10) years, 37% women, who were acutely hospitalized because of stroke or TIA between November 2016 and December 2018. Inclusion criteria were patients aged 18 or above and the major eligibility criteria were the ability to perform the 6-Minute Walking Test (6MWT, meters).

Intervention: The intervention group received standard care plus daily mobile phone text messages (SMS) with instructions to perform regular outdoor walking and functional leg exercises in combination with step counting and training diaries. The control group received standard care.

Outcome measures: Multivariate analysis was performed and age, sex, group allocation, comorbidity, baseline 6MWT, BMI, cognition, and chair-stand tests were entered as possible determinants for changes in 6MWT.

Results: Multiple regression analyses showed that age (Standardized Beta -0.33, 95% CI -3.8 to -1.05, P<0.001), sex (-0.25, 95% CI -68.8 to -10.6, P = 0.008), comorbidity (-0.15, 95% CI -53.7 to 6.4, P = 0.12), baseline BMI (-0.28, 95% CI -7.8 to -1.6, P = 0.004), baseline 6MWT (-0.56, 95% CI -0.5 to -0.3, P <0.001), and possibly allocation to the SMS group (0.17, 95% CI -2.0 to 52.1, P = 0.07) were associated with changes in 6MWT three months after the stroke event. The regression model described 38% of the variance in changes in 6MWT

For peer review only

Conclusions: Post-hoc regression analyses indicated that younger age, male sex, comorbidity, lower BMI, shorter 6MWT at baseline, and allocation to the SMS group contributed to improvement in walking performance at three months in patients with a recent stroke or TIA. These factors may be important when planning SMS or similar rehabilitation services.

Keywords: stroke, risk factors, rehabilitation medicine, clinical trial

Clinical Trial Registry: ClinicalTrial.gov, number NCT02902367

STRENGTHS AND LIMITATIONS OF THIS STUDY

Study data were drawn from a randomized controlled trial and we used established outcome measures.

The study design calls for precaution with causal inferences.

Our findings cannot be generalized to more disabled community-living individuals after a stroke or to individuals with chronic stroke.

The study is relatively small, making the study prone to bias, and all patients are from a single center in Sweden, therefore reducing the generalizability of the results.

For peer review only

Introduction

Approximately three out of four stroke incidents can be attributed to behavioral risk factors—for example, unhealthy diets and sedentary lifestyles (1, 2). Individuals after a stroke are predisposed to functional limitations and a sedentary lifestyle, contributing to the risk of recurrent stroke or cardiovascular complications (1, 3).

Outdoor walking is cost-effective and generally easy to perform. A review showed an average of 4000 steps in the chronic phase after stroke, which is far below the recommended 10,000 steps a day to meet the guidelines for physical activity (3, 4). In high-functioning individuals with stroke, physical activity including walking is low, although we know that walking after discharge from the hospital is important for secondary prevention, minimizing disability, and promoting long-term metabolic health (5). Actions to increase walking performance soon after a stroke or TIA are needed.

In the STROKEWALK study, a previously-reported randomized controlled trial, we found that individuals receiving text messages for three months after stroke and TIA coupled with add-on interventions using a training diary and step counts improved walking and chair-rising performance (6). The present study aims to further study how various baseline characteristics including cardio-metabolic risk markers and group assignment relate to changes in the 6-minute walk test (6MWT), using secondary analyses of data from the STROKEWALK study.

Methods

Study design

For peer review only

This is a post-hoc analysis of the STROKEWALK randomized controlled study performed at the Uppsala University Hospital in Sweden. Determination of sample size, recruitment, data collection, and random allocation procedures have been previously described in detail (6). Ethical approval was obtained from the regional Ethical Review Board of Uppsala University Hospital, Sweden: Dnr: 2015/550. The present study report follows the Strengthening the Reporting of Observational Studies in Epidemiology (STROBE) guidelines for reporting cross-sectional studies (7) and written consent was obtained from all participants.

Recruitment was initiated in November 2016, and the last three-month follow-up assessment was performed in December 2018. Included were participants aged 18 or above with TIA or verified stroke (infarction or intracerebral hemorrhage with first or recurrent event) with sufficient cognition (Montreal Cognitive Assessment scale, MoCA ≥26 points), general disability (modified Rankin Scale ≥2), and good enough walking performance; i.e., ability to perform the six-minute walking test (with or without a walking aid) (6). The exclusion criteria were known subarachnoid hemorrhage, medical problems such as uncontrolled hypertension, untreated arrhythmias, unstable cardiovascular conditions, a dementia diagnosis, severe aphasia, severe psychiatric problems, or cognitive impairment with difficulties understanding instructions (6).

Study outcomes

The 6MWT was used to measure the maximal walking distance during 6 minutes over a 30 -meter course. Changes are described as differences in walking distance at three months(8). The modified Rankin scale was used to assess general disability and is scored from 0 (no symptoms) to 6 (dead) (9). Cognitive function at baseline was assessed using the

For peer review only

Montreal Cognitive Assessment scale (0–30 points),(10) with a higher value indicating better function. The Charlson Comorbidity Index (CCI) was used to classify comorbid conditions (11).

For the chair-stand test, the participant was instructed to rise from a seated position without support as quickly as possible five times in a row (12). The tests were performed with standardized instructions from the Short Physical Performance Battery. The 10-meter walk test was used to measure comfortable walking speed (13).

From the patient's medical records, cardio-metabolic biochemical risk factors such as total, LDL, and HDL cholesterol, C-reactive protein (CRP), and triglycerides were analyzed at hospital admission and diagnoses such as diabetes, hypertension, hypercholesterolemia, and cardiac heart failure were registered (14). Biochemical analyses were performed by accredited methods of the Clinical Chemistry Laboratory at the Uppsala University Hospital, Uppsala. Glycated hemoglobin (HbA1C) was analyzed using non-fasting venous blood samples.

The last registration of supine blood pressure was recorded manually before discharge from the hospital. Smoking and education levels were assessed by yes or no answers to the questions: 'Are you a smoker at this time of your life?' and 'Do you have a university degree?' Body mass index was calculated as body weight (kg) divided by height (m) squared. Weight was recorded with participants wearing light indoor clothing. Height was measured to the nearest cm. A hip-mounted pedometer at weeks 1 and 12 was used for daily step count by individuals in the SMS group (Yamax, SW-200).

The SMS intervention group

The SMS-intervention group received daily text SMS (no cost for the participants) as an addition to standard care with simple instructions on what and how to exercise for three months. The intervention in the SMS-group was comprised of three different strategies: 1) three months of daily SMS-text messages, 2) training diaries, and 3) pedometers for step counts for the first and last week of intervention (6). The text messages gave instructions on how to exercise to increase walking endurance and strength of the lower body, without the possibility to text back for help or advice.

The control group

Patients in the control group were given standard stroke unit care. They had no restrictions regarding physical activity, exercise, or taking part in rehabilitation services, and were given standard recommendations. The control group did not use pedometers since it was considered to be a part of the intervention.

Statistics

In the STROKEWALK study, a sample size of 80 individuals (including a possible drop-out rate of 20%) was determined to provide secure power to detect a 34-meter clinically relevant mean difference in the 6-minute walking test (6). An intention-to-treat analysis was applied for all missing values (dropouts), and the change was assumed to be zero. Thus, follow-up data for dropouts were registered with a baseline carry-forward approach. Descriptive data are reported as means (SD) and medians (IQR). According to the histogram, normal Q–Q plots, and the Kolmogorov–Smirnov test, data on changes in 6MWT were normally distributed. Case-wise diagnostics and standardized residuals were used to identify potential outliers. The Chi-square test, Mann-Whitney *U*-test, and the student's independent test were used to examine baseline differences between those improved at ≥34 meters or <34 meters in 6MWT.

For peer review only

In the regression analyses, explanatory variables for changes in 6MWT were first identified by correlation and univariate regression analyses ($P < 0.05$). Correlation strength was calculated using Spearman´s rho or a Pearson correlation. The identified variables were checked for multi-collinearity by correlation analysis and cross-tabulation and if the correlation coefficient was 0.80 or more the variable with the lowest *r* in relation to the dependent variable was omitted from further regression analysis. The baseline 10-meter walk test was omitted from further analyses due to multi-collinearity. Multiple linear regression analyses were then conducted with the remaining variables to discover which had the greatest impact on changes in walking performance. The ordinal explanatory variable Charlson Comorbidity Index was dichotomized and grouped to "no comorbidity" or "one or more than one comorbidity". The ordinal explanatory variable Montreal Cognitive Assessment scale was dichotomized and grouped to the cutoff score ≥26 points. Changes in 6MWT were used as the dependent variable. We adjusted for age, sex, comorbidity and intervention or control group allocation. Case-wise diagnostics showed that one individual could be considered an outlier; i.e., with an increase in 6MWT of 365 meters, but were not omitted from further analysis.

Statistical significance was set at a *P* value <0.05. The Statistical Package for the Social Sciences (SPSS), version 25, was used for the analyses (SPSS Inc., Chicago, IL, U.S.A.).

Results

Seventy-nine patients with a mean age 63.9 (10.4) years, 29 women, mean BMI of 27.5 (4.5) kg/m^2 were enrolled and allocated to either SMS intervention (n =40) or control group (n =39).(6) Assessments were performed with a median of five (IQR 6) days after stroke or

For peer review only

TIA and after three months. At baseline assessments, seven individuals temporarily used a walking aid.

Table 1 gives the clinical characteristics for all individuals at baseline and changes in 6MWT.

---*Insert Table 1 about here*---

At baseline, 27% of participants had a BMI ≥30 (obesity), 43% had a BMI between 25 and 29.9 (overweight), and 30% had a BMI <25. In this study, all participants could perform the 6MWT on both occasions and no adverse advents occurred during testing. The mean 6MWT was 480 (105) meters. At three months, the mean 6MWT was 523 (104) meters. The mean change in 6MWT was 55 (71) and 33 (80) meters for the SMS and control groups, respectively (P =0.2). On average, the participants in the SMS group walked 6335 steps per day in the first week of intervention and 8173 steps per day after three months, an increase of 29% (n =33).

Linear regression analyses for identification of factors related to change in walking capacity

Table 2 show correlations of possible variables for the regression models.

---*Insert Table 2 about here*---

The differences in walking performance were significantly associated in a univariate analysis with baseline BMI and 6MWT at baseline (Table 3). After adjusting for age, sex, comorbidity, and group assignment, the final model still included baseline 6MWT and BMI,

For peer review only

which together with age, sex, and group assignment explained 38% of the variance (Table 3). Younger individuals, men, the SMS intervention group, and those with lower baseline BMI and shorter 6MWT at baseline were more likely to improve their walking performance.

---Insert Table 3 about here---

Discussion

In this post-hoc study, we showed in regression analyses that younger age, male sex, SMS assignment, lower baseline BMI, and less distance walked in 6MWT significantly predicted positive change in 6MWT three months after stroke or TIA.

In our study sample, those with higher age improved less in the 6MWT at three months. Thus, our finding is in line with a general tendency to be less physically active at older ages (14). Age-related physiological changes like reduced oxygen uptake capacity (VO_2 max), changes in heart and lungs, loss of muscle mass and strength, more comorbidity, and medications in older age may affect the intensity and ability to perform outdoor walking in the present study(1, 15). However, each individual in this study could find their own suggested intensity level by using the Borg scale.

In the present study, about 70% of the participants had a BMI above 25. Higher BMI predicted less improvement in walking distance as measured with the 6MWT. It can be speculated that those with obesity also were sarcopenic; i.e., displayed sarcopenic obesity, which is known to affect walking performance. However, we did not collect data on muscle mass in this study. In our study sample, we found weight fluctuation in both directions after three months, which might have affected the results (14). A deterioration in health might be seen in individuals with high BMI due to difficulties being active in daily living. This study

For peer review only

indicates that individuals after a stroke and with obesity need help initiating lifestyle changes to increase physical activity.

In a longitudinal study of cardiovascular disease secondary prevention, an inverse association between walking speed and mortality was found; with a 53% reduction in mortality risk for those with the highest walking speed (3.8–6.2 km/h). In a longitudinal study of cardiovascular disease secondary prevention, an inverse association between walking speed and mortality was found; with a 53% reduction in mortality risk for those with the highest walking speed (3.8–6.2 km/h) (16). In contrast to the present study, the participants were all women, but they had a similar risk factor profile (16). Another cross-sectional study, using a population three months or longer after stroke found that balance as measured with Berg Balance Scale was a significant predictor of free-living walking activity, and explained 13% of the variance (17). In our selected sample of individuals with high motor functions at baseline, balance was not a major problem (6).

Fini et al. reported a mean of 4078 steps per day, six months or more after stroke.(18) The number of steps we found after the first week of SMS intervention in our sample with recent stroke and TIA is higher than that, averaging 6335 steps per day the first week of intervention and a further increase of 29% after three months. In older adults, a dose–response relationship has been observed for sedentary behavior, as well as between steps per day and mortality(19). Walking speed, steps, and distance can reflect functional status and health and is important for activities and community ambulation in daily life (13). Furthermore, this study sample included more males which could have an impact on the results since male sex was associated with greater improvement in walking performance. In a larger study (AVERT trial) with 2100 participants which examined factors associated with achievement of independent walking at three months after stroke, sex was not an associated factor (20).

For peer review only

The reason why those that walked shorter distances at baseline improved the most is unknown, but regression towards the mean cannot be excluded. Still, in those with the poorest walking performance a smaller increase in steps may be sufficient to have a positive impact on health (21). New epidemiological studies measuring physical activity with accelerometers show that the positive effects of physical activity may have been underestimated (21). A large prospective study indicated that up to 10,000 steps a day was associated with a lower risk of cardiovascular incidence and mortality (21). Additional risk reduction was also found with steps performed at a higher intensity and there was no minimum threshold for the association between increasing steps per day with morbidity and mortality (21). This can be used to motivate the least active individuals to increase their outdoor walking and number of steps per day.

Limitations and strengths

Some methodological issues need to be addressed in this study. One limitation is that the study design calls for precaution with causal inferences. Another limitation is that our findings cannot be generalized to more disabled community-living individuals after a stroke or to individuals with chronic stroke. However, since we included participants at the hospital with different socio-economic statuses and educational backgrounds, we believe this sample to be representative of the acute stroke and TIA population with fewer motor deficits. Finally, the study is relatively small, making the study prone to bias, and all patients are from a single center in Sweden, therefore reducing the generalizability of the results.

One strength of the present study is that study data were drawn from a randomized controlled trial and that we used established outcome measures.

For peer review only

Conclusion: In summary, younger age, male sex, comorbidity, lower BMI, shorter 6MWT at baseline, and allocation to the SMS group contributed most to improvement in walking performance in patients with a recent stroke or TIA. These factors may be important to consider when planning SMS or similar rehabilitation services. Cost-effective and easy delivered interventions for individuals with minor stroke or TIA still requires further targeted research.

Author affiliations:

Birgit Vahlberg, Ph.D., RPT Department of Public Health and Caring Sciences, Geriatrics, Uppsala University, Uppsala, Sweden.

Staffan Eriksson, Ph.D., RPT Department of Public Health and Caring Sciences, Geriatrics, Uppsala University, Uppsala, and Centre for Clinical Research, Sörmland, Uppsala University, Eskilstuna, Sweden.

Ulf Holmbäck, Associate Professor, Department of Public Health and Caring Sciences, Clinical Nutrition and Metabolism, Uppsala University, Uppsala, Sweden.

Tommy Cederholm, Professor, MD, Department of Public Health and Caring Sciences, Clinical Nutrition and Metabolism, Uppsala University, Uppsala, Sweden.

Erik Lundström, Professor, MD, Department of Medical Sciences, Neurology, Uppsala University, Sweden.

Contributors: This study was conceived, organized and managed by BV, SE, UH, TC and EL. BV acts as a guarantor of the study. All authors listed above contributed to the study

For peer review only

design and data interpretation. Writing of the first draft of the paper was done by BMV and all authors were involved in preparation and critique of the manuscript and reviewed the paper prior to submission.

Funding: This study was funded by the Medical Faculty at Uppsala University, the Swedish Stroke Association (Stroke-Riksförbundet), and the Geriatric funding, Sweden.

Patient consent for publication: Not applicable.

Patient and Public Involvement Statement: A previous pilot study were conducted to test the design of the current study.

Competing interests: The author(s) report that there are no competing interests to declare.

Ethics approval: This study involves human participants and was approved by the regional Ethical Review Board of Uppsala University Hospital, Sweden: Dnr: 2015/550.

Data availability statement: The data set associated with this work is available from the corresponding author upon reasonable request.

ORCiD iDs

BV: http://orcid.org/0000-0002-1508-1435

SE: http://orcid.org/0000-0003-3572-8918

For peer review only

UH: http://orcid.org/0000-0002-0764-5878

TC: http://orcid.org/0000-0002-0764-0725

EL: http://orcid.org/0000-0002-5313-9052

Figure: None.

For peer review only

References

1. Virani SS, Alonso A, Aparicio HJ, Benjamin EJ, Bittencourt MS, Callaway CW, et al. Heart Disease and Stroke Statistics-2021 Update: A Report From the American Heart Association. Circulation. 2021;143(8):e254-e743.

2. RIKSstroke. Stroke och TIA. Riksstrokes årsrapport från 2019 års data. Umeå; Riksstroke 2020. available from:htpp://www.riksstroke.org/wp-content/uploads/2020/09/Riksstroke_Årsrapport-2019_slutversionWEB-1.pdf; (cited 15 May, 2021); .

3. English C, Manns PJ, Tucak C, Bernhardt J. Physical activity and sedentary behaviors in people with stroke living in the community: a systematic review. Phys Ther. 2014;94(2):185-96.

4. Tudor-Locke C, Bassett DR. How many steps/day are enough? Preliminary pedometer indices for public health. Sports Med. 2004;34(1):1-8.

5. Saunders DH, Sanderson M, Hayes S, Kilrane M, Greig CA, Brazzelli M, et al. Physical fitness training for stroke patients. Cochrane Database Syst Rev. 2016;3:CD003316.

6. Vahlberg B, Lundström E, Eriksson S, Holmbäck U, Cederholm T. Effects on walking performance and lower body strength by short message service guided training after stroke or transient ischemic attack (The STROKEWALK Study): a randomized controlled trial. Clin Rehabil. 2021;35(2):276-87.

7. Cuschieri S. The STROBE guidelines. Saudi J Anaesth. 2019;13(Suppl 1):S31-S4.

8. Brooks D, Solway S, Gibbons W. ATS statement on six-minute walk test. Am J Respir Crit Care Med. 2003;167(9):1287.

For peer review only

9. Banks JL, Marotta CA. Outcomes validity and reliability of the modified Rankin scale: implications for stroke clinical trials: a literature review and synthesis. Stroke. 2007;38(3):1091-6.

10. Nasreddine ZS, Phillips NA, Bédirian V, Charbonneau S, Whitehead V, Collin I, et al. The Montreal Cognitive Assessment, MoCA: a brief screening tool for mild cognitive impairment. J Am Geriatr Soc. 2005;53(4):695-9.

11. Goldstein LB, Samsa GP, Matchar DB, Horner RD. Charlson Index comorbidity adjustment for ischemic stroke outcome studies. Stroke. 2004;35(8):1941-5.

12. Guralnik JM, Simonsick EM, Ferrucci L, Glynn RJ, Berkman LF, Blazer DG, et al. A short physical performance battery assessing lower extremity function: association with self-reported disability and prediction of mortality and nursing home admission. J Gerontol. 1994;49(2):M85-94.

13. Studenski S, Perera S, Patel K, Rosano C, Faulkner K, Inzitari M, et al. Gait speed and survival in older adults. JAMA. 2011;305(1):50-8.

14. Vahlberg BM, Lundström E, Eriksson S, Holmback U, Cederholm T. Potential effects on cardiometabolic risk factors and body composition by short message service (SMS)-guided training after recent minor stroke or transient ischaemic attack: post hoc analyses of the STROKEWALK randomised controlled trial. BMJ Open. 2021;11(10):e054851.

15. Cruz-Jentoft AJ, Bahat G, Bauer J, Boirie Y, Bruyère O, Cederholm T, et al. Sarcopenia: revised European consensus on definition and diagnosis. Age Ageing. 2019;48(4):601.

16. Raisi A, Zerbini V, Myers J, Masotti S, Piva T, Lordi R, et al. Moderate walking speed and survival association across 23-years follow-up in female patients with cardiovascular disease. Int J Cardiol. 2023;371:371-6.

For peer review only

404 17. van de Port I, Punt M, Meijer JW. Walking activity and its determinants in free-living ambulatory people in a chronic phase after stroke: a cross-sectional study. Disabil Rehabil. 2020;42(5):636-41.

407 18. Fini NA, Holland AE, Keating J, Simek J, Bernhardt J. How Physically Active Are People Following Stroke? Systematic Review and Quantitative Synthesis. Phys Ther. 2017;97(7):707-17.

410 19. Rojer AGM, Ramsey KA, Trappenburg MC, van Rijssen NM, Otten RHJ, Heymans MW, et al. Instrumented measures of sedentary behaviour and physical activity are associated with mortality in community-dwelling older adults: A systematic review, meta-analysis and meta-regression analysis. Ageing Res Rev. 2020;61:101061.

414 20. Kennedy C, Bernhardt J, Churilov L, Collier JM, Ellery F, Rethnam V, et al. Factors associated with time to independent walking recovery post-stroke. J Neurol Neurosurg Psychiatry. 2021;92(7):702-8.

417 21. Del Pozo Cruz B, Ahmadi MN, Lee IM, Stamatakis E. Prospective Associations of Daily Step Counts and Intensity With Cancer and Cardiovascular Disease Incidence and Mortality and All-Cause Mortality. JAMA Intern Med. 2022;182(11):1139-48.

420

421

For peer review only

Table 1. Baseline characteristics of all patients in the study with ≥34 meters increase in the six-minute walking test at three months versus patients with < 34 meters increase in the six-minute walking test.

	**Study population**	**Changes in the 6-minute walking test**			
	**Baseline**	**≥ 34 m (n=38)**	**<34 m (n=41)**	**P-value**	**Missing values, n (%)**
Age, mean (SD)	63.9 (10.4)	61.9 (9.1)	65.7 (11.2)	0.01	0
Female, n (%)	29 (36.7)	11 (28.9)	18 (43.9)	0.17	0
SMS group, n (%)	40 (50.6)	26 (65.0)	14 (35.0)	0.002	0
Control group, n (%)	39 (49.4)	12 (30.8)	27 (69.2)		
modified Rankin Scale, 0-2				0.47	0
0	11 (13.9)	6 (54.5)	5 (45.5)		
1	53 (67.1)	23 (43.4)	30 (56.6)		
2	15 (19.0)	9 (60.0)	6 (40.0)		
Diagnosis, n (%)				0.70	0

Cerebral infarction	66 (83.5)	29 (50.9)	28 (49.1)		
Intracerebral hemorrhage	9 (11.4)	4 (44.4)	5(55.6)		
TIA	13 (16.5)	8 (61.5)	5 (38.5)		
Charlson Comorbidity Index, n (%)					0
No comorbidity	47 (59.5)	25 (53.2)	22 (46.8)	0.27	
≥1	32 (40.5)	13 (40.6)	19 (59.4)		
BMI, mean (SD)	27.5 (4.5)	26.55 (3.77)	28.39 (5.03)	0.14	0
Diabetes mellitus-2, yes n (%)	12 (15.2)	5 (13.2)	7 (17.1)	0.63	0
University studies, yes n (%)	40 (62)	16 (42.1)	24 (58.5)	0.14	0
Non-smoking, n (%)	71 (90)	33 (86.8)	38 (92.7)	0.39	0
Step counts (SMS group), mean (SD)	6335 (2747)	6612 (2741)	5757 (2786)	0.61	3 (7.5)
SGPALS, n (%)					0
Sedentary	11 (13.9)	6 (54.5)	5 (45.5)	0.47	
Light Physical Activity	53 (67.1)	23 (43.4)	30 (56.6)		
Moderate/High Physical Activity	15 (19.0)	9 (60.0)	6 (40.0)		
P-HDL cholesterol, (mmol/l), mean (SD)	1.32 (0.39)	1.35 (0.45)	1.26 (0.32)	0.44	3 (3.8)

For peer review only

P-LDL cholesterol, (mmol/l), mean (SD)	3.16 (1.09)	3.18 (1.16)	3.13 (1.04)	0.86	3 (3.8)
P-Cholesterol, (mmol/l), mean (SD)	5.13 (1.20)	5.16 (1.30)	5.13 (1.12)	0.86	3 (3.8)
P-Triglycerides, (mmol/l), mean (SD)	1.35 (0.63)	1.20 (0.46)	1.50 (0.74)	0.03	3 (3.8)
P-HbA1C, mmol/mol), mean (SD)	38.0 (7.54)	37.73 (8.33)	38.26 (6.77)	0.47	3 (3.8)
P-C reactive protein (mg/L), mean (SD)	4.92 (19.40)	6.80 (27.80)	3.20 (3.95)	0.20	6 (7.6)
P-Creatinine (mmol/L), mean (SD)	85.12 (25.53)	90.80	78.97 (17.83)	0.11	2 (2.6)
SBP, (mm HG), mean (SD)	130.63 (16.56)	129.8 (16.17)	131.36 (17.09)	0.81	4 (5)
DBP, (mm HG), mean (SD)	76.08 (11.0)	79.17 (11.24)	73.23 (10.09)	0.02	4 (5)

Abbreviations: BMI, body mass index, CRP, C-reactive protein; DBP, diastolic blood pressure; HbA1C, glycated hemoglobin; HDL, high-density lipoprotein; LDL, low-density lipoprotein; SGPALS, Saltin Grimby Physical Activity Scale; SMS, short message service group; SBP, systolic blood pressure

For peer review only

Table 2. Correlation of the variables used in the regression models.

	Change 6-minute walking test	P-value
Age (years)	-0.203	0.07
Sex (female)	-0.127	0.26
Charlson Comorbidity Index (≥1 comorbidity)	-0.198	0.08
Group (SMS/control)	0.237*	0.036
BMI, (kg/m^2)	-225*	0.046
6-minute walking test, baseline (meters)	-0.38**	<0.001
Chair-stand test, (seconds)	0.194	0.086
Montreal Cognitive Assessment, (≥26 points)	-0.034	0.78
Saltin Grimby Physical Activity Level Scale (Sedentary, light physical activity, moderate/high physical activity	0.078	0.49

For peer review only

Table 3. Univariate and multivariate regression analyses with changes in walking performance (6-minute walking test) as the dependent variable and age, sex, group allocation, comorbidity, baseline BMI-, chair-stand test-, cognition-, and 6-minute walk test as explanatory variables in individuals after stroke and TIA.

	Univariate analysis				Multivariate analysis		
	**Beta standardized**	**Adjusted R square**	**95% CI**	**P**	**Beta standardized**	**95% CI**	**P**
Age, yrs	-203	0.029	-3.1 to 0.1	0.073	-0.33	-3.8 to -1.05	<0.001
Sex, female	-0.121	0.002	-54.4 to 16.4	0.29	-0.25	-68.8 to -10.6	0.008
CCI, ≥1 comorbidity	-0.19	0.024	-63.8 to 4.9	0.09	-0.15	-53.7 to 6.4	0.12
Group, SMS	0.145	0.008	-12.1 to 55.9	0.20	0.17	-2.0 to 52.1	0.07
BMI, kg/m^2	-0.225	0.038	-7.5 to -0.07	0.046	-0.28	-7.8 to –1.6	0.004
6-minute walk test, meters	-0.38	0.134	-0.4 to -0.1	<0.001	-0.56	-0.5 to -0.3	<0.001

Abbreviations: CCI, Charlson Comorbidity Index; SMS, short message service; MoCA, Montreal Cognitive Assessment

The adjusted R square for the multivariate analysis was 0.38.

For peer review only

STROBE Statement—checklist of items that should be included in reports of observational studies

	Item No	Recommendation
**Title and abstract**	1	(*a*) Indicate the study's design with a commonly used term in the title or the abstract
		(*b*) Provide in the abstract an informative and balanced summary of what was done and what was found
**Introduction**		
Background/rationale	2	Explain the scientific background and rationale for the investigation being reported
Objectives	3	State specific objectives, including any prespecified hypotheses
**Methods**		
Study design	4	Present key elements of study design early in the paper
Setting	5	Describe the setting, locations, and relevant dates, including periods of recruitment, exposure, follow-up, and data collection
Participants	6	(*a*) *Cohort study*—Give the eligibility criteria, and the sources and methods of selection of participants. Describe methods of follow-up<br>*Case-control study*—Give the eligibility criteria, and the sources and methods of case ascertainment and control selection. Give the rationale for the choice of cases and controls<br>*Cross-sectional study*—Give the eligibility criteria, and the sources and methods of selection of participants
		(*b*) *Cohort study*—For matched studies, give matching criteria and number of exposed and unexposed<br>*Case-control study*—For matched studies, give matching criteria and the number of controls per case
Variables	7	Clearly define all outcomes, exposures, predictors, potential confounders, and effect modifiers. Give diagnostic criteria, if applicable
Data sources/ measurement	8*	For each variable of interest, give sources of data and details of methods of assessment (measurement). Describe comparability of assessment methods if there is more than one group
Bias	9	Describe any efforts to address potential sources of bias
Study size	10	Explain how the study size was arrived at
Quantitative variables	11	Explain how quantitative variables were handled in the analyses. If applicable, describe which groupings were chosen and why
Statistical methods	12	(*a*) Describe all statistical methods, including those used to control for confounding
		(*b*) Describe any methods used to examine subgroups and interactions
		(*c*) Explain how missing data were addressed
		(*d*) *Cohort study*—If applicable, explain how loss to follow-up was addressed<br>*Case-control study*—If applicable, explain how matching of cases and controls was addressed<br>*Cross-sectional study*—If applicable, describe analytical methods taking account of sampling strategy
		(*e*) Describe any sensitivity analyses

Continued on next page

For peer review only - http://bmjopen.bmj.com/site/about/guidelines.xhtml

**Results**		
Participants	13*	(a) Report numbers of individuals at each stage of study—eg numbers potentially eligible, examined for eligibility, confirmed eligible, included in the study, completing follow-up, and analysed
		(b) Give reasons for non-participation at each stage
		(c) Consider use of a flow diagram
Descriptive data	14*	(a) Give characteristics of study participants (eg demographic, clinical, social) and information on exposures and potential confounders
		(b) Indicate number of participants with missing data for each variable of interest
		(c) *Cohort study*—Summarise follow-up time (eg, average and total amount)
Outcome data	15*	*Cohort study*—Report numbers of outcome events or summary measures over time
		Case-control study—Report numbers in each exposure category, or summary measures of exposure
		Cross-sectional study—Report numbers of outcome events or summary measures
Main results	16	(*a*) Give unadjusted estimates and, if applicable, confounder-adjusted estimates and their precision (eg, 95% confidence interval). Make clear which confounders were adjusted for and why they were included
		(*b*) Report category boundaries when continuous variables were categorized
		(*c*) If relevant, consider translating estimates of relative risk into absolute risk for a meaningful time period
Other analyses	17	Report other analyses done—eg analyses of subgroups and interactions, and sensitivity analyses
**Discussion**		
Key results	18	Summarise key results with reference to study objectives
Limitations	19	Discuss limitations of the study, taking into account sources of potential bias or imprecision. Discuss both direction and magnitude of any potential bias
Interpretation	20	Give a cautious overall interpretation of results considering objectives, limitations, multiplicity of analyses, results from similar studies, and other relevant evidence
Generalisability	21	Discuss the generalisability (external validity) of the study results
**Other information**		
Funding	22	Give the source of funding and the role of the funders for the present study and, if applicable, for the original study on which the present article is based

*Give information separately for cases and controls in case-control studies and, if applicable, for exposed and unexposed groups in cohort and cross-sectional studies.

Note: An Explanation and Elaboration article discusses each checklist item and gives methodological background and published examples of transparent reporting. The STROBE checklist is best used in conjunction with this article (freely available on the Web sites of PLoS Medicine at http://www.plosmedicine.org/, Annals of Internal Medicine at http://www.annals.org/, and Epidemiology at http://www.epidem.com/). Information on the STROBE Initiative is available at www.strobe-statement.org.

For peer review only

BMJ Open

Factors associated with changes in walking performance in individuals three months after stroke or TIA—secondary analyses from a randomized controlled trial of SMS-delivered training instructions in Sweden

Journal:	*BMJ Open*
Manuscript ID	bmjopen-2023-078180.R1
Article Type:	Original research
Date Submitted by the Author:	05-Dec-2023
Complete List of Authors:	Vahlberg, Birgit; Uppsala University, Department of Public Health and Caring Sciences, Geriatrics<br>Eriksson, Staffan; Uppsala University, Department of Public Health and Caring Sciences, Uppsala University; Uppsala University, Centre for Clinical Research, Sörmland, Eskilstuna;<br>Holmbäck, Ulf; Uppsala University, Department of Public Health and Caring Sciences, Clinical Nutrition and Metabolism,<br>Lundström, Erik; Uppsala University, Department of Medical sciences, Neurology
**Primary Subject Heading**:	Rehabilitation medicine
Secondary Subject Heading:	Neurology, Geriatric medicine, Public health
Keywords:	Stroke < NEUROLOGY, Risk Factors, REHABILITATION MEDICINE, Clinical Trial

SCHOLARONE™ Manuscripts

I, the Submitting Author has the right to grant and does grant on behalf of all authors of the Work (as defined in the below author licence), an exclusive licence and/or a non-exclusive licence for contributions from authors who are: i) UK Crown employees; ii) where BMJ has agreed a CC-BY licence shall apply, and/or iii) in accordance with the terms applicable for US Federal Government officers or employees acting as part of their official duties; on a worldwide, perpetual, irrevocable, royalty-free basis to BMJ Publishing Group Ltd ("BMJ") its licensees and where the relevant Journal is co-owned by BMJ to the co-owners of the Journal, to publish the Work in this journal and any other BMJ products and to exploit all rights, as set out in our licence.

The Submitting Author accepts and understands that any supply made under these terms is made by BMJ to the Submitting Author unless you are acting as an employee on behalf of your employer or a postgraduate student of an affiliated institution which is paying any applicable article publishing charge ("APC") for Open Access articles. Where the Submitting Author wishes to make the Work available on an Open Access basis (and intends to pay the relevant APC), the terms of reuse of such Open Access shall be governed by a Creative Commons licence – details of these licences and which Creative Commons licence will apply to this Work are set out in our licence referred to above.

Other than as permitted in any relevant BMJ Author's Self Archiving Policies, I confirm this Work has not been accepted for publication elsewhere, is not being considered for publication elsewhere and does not duplicate material already published. I confirm all authors consent to publication of this Work and authorise the granting of this licence.

For peer review only

Factors associated with changes in walking performance in individuals three months after stroke or TIA—secondary analyses from a randomized controlled trial of SMS-delivered training instructions in Sweden

Short title: Predicting changes in walking performance three months after recent stroke and TIA in Sweden.

Birgit Vahlberg, PhD, RPT, Department of Public Health and Caring Sciences, Geriatrics, Uppsala University, Uppsala.

Staffan Eriksson, PhD, RPT, Department of Public Health and Caring Sciences, Geriatrics, Uppsala University, Uppsala; Centre for Clinical Research, Sörmland, Uppsala University, Eskilstuna.

Ulf Holmbäck, Associate Professor, Nutritionist, Department of Public Health and Caring Sciences, Clinical Nutrition and Metabolism, Uppsala University, Uppsala.

Erik Lundström, Professor, MD, Department of Medical Sciences, Neurology, Uppsala University, Uppsala.

All authors are from Sweden.

Corresponding author: Birgit Vahlberg

Address: Department of Public Health and Caring Sciences, Geriatrics,

Uppsala University, BMC A9

S-751 24 Uppsala, Sweden

Telephone: +46 709583473

E-mail: Birgit.Vahlberg@pubcare.uu.se

Word count main text: 3433

Keywords: stroke, risk factors, rehabilitation medicine, clinical trial

For peer review only

ABSTRACT:

Objectives: To identify factors related to changes in walking performance in individuals 3 months after a stroke or TIA.

Design: Cross-sectional study with post-hoc analysis of a randomized controlled study.

Setting: University Hospital, Sweden.

Participants: 79 individuals, 64 (10) years, 37% women, who were acutely hospitalized because of stroke or TIA between November 2016 and December 2018. Inclusion criteria were patients aged 18 or above and the major eligibility criteria were the ability to perform the 6-minute walking test.

Intervention: The intervention group received standard care plus daily mobile phone text messages (SMS) with instructions to perform regular outdoor walking and functional leg exercises in combination with step counting and training diaries. The control group received standard care.

Outcome measures: Multivariate analysis was performed and age, sex, group allocation, comorbidity, baseline 6-minute walk test, BMI, cognition, and chair-stand tests were entered as possible determinants for changes in the 6-minute walk test.

Results: Multiple regression analyses showed that age (Standardized Beta -0.33, 95% CI -3.8 to -1.05, P<0.001), sex (-0.24, 95% CI -66.9 to -8.0, P = 0.014), no comorbidity (-0.16, 95% CI -55.5 to 5.4, P = 0.12), baseline BMI (-0.29, 95% CI -8.1 to -1.6, P = 0.004), baseline 6-minute walk test (-0.55, 95% CI -0.5 to -0.3, P <0.001) were associated with changes in 6-minute walk test three months after the stroke event. The regression model described 36% of the variance in changes in the 6-minute walk test.

For peer review only

Conclusions: Post-hoc regression analyses indicated that younger age, male sex, lower BMI, and shorter 6-minute walk test at baseline and possible no comorbidity contributed to improvement in walking performance at three months in patients with a recent stroke or TIA. These factors may be important when planning secondary prevention actions.

Clinical Trial Registry: ClinicalTrial.gov, number NCT02902367

STRENGTHS AND LIMITATIONS OF THIS STUDY

Study data were drawn from a randomized controlled trial and we used established outcome measures.

The study design calls for precaution with causal inferences.

Our findings cannot be generalized to more disabled community-living individuals after a stroke or to individuals with chronic stroke.

The study is relatively small, making the study prone to bias, and all patients are from a single center in Sweden, therefore reducing the generalizability of the results.

For peer review only

Introduction

Approximately three out of four stroke incidents can be attributed to behavioral risk factors—for example, unhealthy diets and sedentary lifestyles including low levels of physical activity (PA) (1, 2). Individuals after a stroke are predisposed to functional limitations and a sedentary lifestyle, contributing to the risk of recurrent stroke or cardiovascular complications (1, 3).

Outdoor walking is cost-effective and generally easy to perform. A review showed an average of 4000 steps in the chronic phase after stroke, which is far below the recommended 10,000 steps a day to meet the guidelines for physical activity (3, 4). In high-functioning individuals with stroke, physical activity including walking is low, although we know that walking after discharge from the hospital is important for secondary prevention, minimizing disability, and promoting long-term metabolic health (5, 6). Walking ability and better balance are associated with higher PA levels in everyday life after stroke and lower mood is related to low PA in people with chronic stroke (6, 7). Hence, in the work with secondary prevention, actions to increase walking performance soon after a stroke or TIA are needed, and, in this work knowledge about factors associated with changes in walking distance is important (8). Both stroke and TIA indicate ongoing arteriosclerotic changes in the vessels that can lead to further cardiovascular events and PA is known to decrease the risk of stroke, TIA, and myocardial infarction (9-11). There is a lack of studies investigating changes in walking performance in high-functioning individuals soon after stroke and TIA, and the few studies tend to be conducted months after stroke with a narrow focus on physical functions, overlooking cognition and cardiometabolic risk markers (8, 12).

For peer review only - http://bmjopen.bmj.com/site/about/guidelines.xhtml

In the STROKEWALK study, a previously-reported randomized controlled trial, we found that individuals receiving text messages for three months after stroke and TIA coupled with add-on interventions using a training diary and step counts improved walking distance and chair-rising performance (13). The present study aims to further study how various baseline characteristics including cardio-metabolic risk markers relate to changes in the 6-minute walk test, using secondary analyses of data from the STROKEWALK study.

Methods

Study design

This is a post-hoc analysis of the STROKEWALK randomized controlled study performed at the Uppsala University Hospital in Sweden. Determination of sample size, recruitment, data collection, and random allocation procedures have been previously described in detail (13). Ethical approval was obtained from the regional Ethical Review Board of Uppsala University Hospital, Sweden: Dnr: 2015/550. The present study report follows the Strengthening the Reporting of Observational Studies in Epidemiology (STROBE) guidelines for reporting cross-sectional studies (14) and written consent was obtained from all participants.

Recruitment was initiated in November 2016, and the last three-month follow-up assessment was performed in December 2018. Included were participants aged 18 or above with TIA or verified stroke (infarction or intracerebral hemorrhage with first or recurrent event) with sufficient cognition (Montreal Cognitive Assessment scale, MoCA ≥26 points), general disability (modified Rankin Scale ≥2), and good enough walking performance; i.e., ability to perform the six-minute walking test (with or without a walking aid) (13). The exclusion criteria were known subarachnoid hemorrhage, medical problems such as uncontrolled

For peer review only - http://bmjopen.bmj.com/site/about/guidelines.xhtml

For peer review only

hypertension, untreated arrhythmias, unstable cardiovascular conditions, a dementia diagnosis, severe aphasia, severe psychiatric problems, or cognitive impairment with difficulties understanding instructions (13).

Study outcomes

The 6-minute walk test was used to measure the maximal walking distance during six minutes over a 30-meter course. Changes are described as differences in walking distance at three months (13, 15).

Baseline assessments

The modified Rankin scale was used to assess general disability and is scored from 0 (no symptoms) to 6 (dead) (13, 16).

Cognitive function at baseline was assessed using the Montreal Cognitive Assessment scale (0–30 points),(13, 17) with a higher value indicating better function.

The Charlson Comorbidity Index (CCI) was used to classify comorbid conditions (13, 18).

The last registration of supine blood pressure was recorded manually before discharge from the hospital. Smoking and education levels were assessed by yes or no answers to the questions: ‘Are you a smoker at this time of your life?’ and ‘Do you have a university degree?’ For the chair-stand test, the participant was instructed to rise from a seated position without support as quickly as possible five times in a row (13, 19). The test was performed with standardized instructions from the Short Physical Performance Battery (13, 20). The chair-stand test was a measure of lower body strength and the severity of the lower limb impairment.

For peer review only - http://bmjopen.bmj.com/site/about/guidelines.xhtml

142 The 10-meter walk test was used to measure comfortable walking speed (13, 21).

143 From the patient's medical records, cardio-metabolic biochemical risk factors such as total, LDL, and HDL cholesterol, C-reactive protein (CRP), and triglycerides were analyzed at hospital admission and diagnoses such as diabetes, hypertension, hypercholesterolemia, and cardiac heart failure were registered (22). Biochemical analyses were performed by accredited methods of the Clinical Chemistry Laboratory at the Uppsala University Hospital, Uppsala. Glycated hemoglobin (HbA1C) was analyzed using non-fasting venous blood samples.

150 Body mass index was calculated as body weight (kg) divided by height (m) squared. Weight was recorded with participants wearing light indoor clothing. Height was measured to the nearest cm. A hip-mounted pedometer at weeks 1 and 12 was used for daily step count by individuals in the SMS group (Yamax, SW-200).

154

155 *The SMS intervention group*

156 The SMS-intervention group received daily text SMS (no cost for the participants) as an addition to standard care with simple instructions on what and how to exercise for three months. The intervention in the SMS group was comprised of three different strategies: 1) three months of daily SMS-text messages, 2) training diaries, and 3) pedometers for step counts for the first and last week of intervention (13). The text messages gave instructions on how to exercise to increase walking endurance and strength of the lower body, without the possibility of texting back for help or advice.

163 *The control group*

view only

Patients in the control group were given standard stroke unit care. They had no restrictions regarding physical activity, exercise, or taking part in rehabilitation services, and were given standard recommendations. The control group did not use pedometers since it was considered to be a part of the intervention.

Patient and Public Involvement Statement: A previous pilot study was conducted to test the design of the randomized controlled trial. The intervention was designed in collaboration between individuals with stroke and TIA, healthcare professionals, and researchers.

Statistics

In the STROKEWALK study, a sample size of 80 individuals (including a possible drop-out rate of 20%) was determined to provide secure power to detect a 34-meter clinically relevant mean difference in the 6-minute walking test (13). An intention-to-treat analysis was applied for all missing values (dropouts), and the change was assumed to be zero. Thus, follow-up data for dropouts were registered with a baseline carry-forward approach. Descriptive data are reported as means (SD) and medians (IQR). According to the histogram, normal Q–Q plots, and the Kolmogorov–Smirnov test, data on changes in the 6-minute walk test were normally distributed. Case-wise diagnostics and standardized residuals were used to identify potential outliers. Differences in step counts from baseline to three months for the SMS group were calculated with the Student´s paired-sample t-test. Baseline differences between those that improved ≥34 meters or <34 meters in the 6-minute walk test were assessed using the Student´s t-test for continuous, normally distributed variables, and the Mann Whitney-U test was applied for ordinal or non-normally distributed variables. The Chi-square test was used for categorical variables. The cut-off of 34 meters was used for power analyses in the original study (13).

For peer review only - http://bmjopen.bmj.com/site/about/guidelines.xhtml

In the regression analyses, baseline explanatory variables for changes in the 6-minute walk test were first identified by correlation and univariate regression analyses ($P < 0 .05$). Correlation strength was calculated using Spearman´s rho for non-parametric data or a Pearson correlation for continuous normally distributed variables. The identified variables were checked for multi-collinearity by correlation analysis and cross-tabulation and if the correlation coefficient was 0.80 or more the variable with the lowest *r* in relation to the dependent variable was omitted from further regression analysis. The baseline 10-meter walk test was omitted from further analyses due to multi-collinearity. Multiple linear regression analyses were then conducted with the remaining variables to discover which had the greatest impact on changes in walking performance. The ordinal explanatory variable Charlson Comorbidity Index was dichotomized and grouped into “no comorbidity” or “one or more than one comorbidity”. The ordinal explanatory variable Montreal Cognitive Assessment scale was dichotomized and grouped to the cutoff score ≥26 points. Changes in 6MWT were used as the dependent variable. We adjusted for age, sex, and comorbidity. Case-wise diagnostics showed that one individual could be considered an outlier; i.e., with an increase in 6-minute walk test of 365 meters, but were not omitted from further analysis. In this study, a sensitivity analysis with complete case analyses was also carried out. The univariate and multivariate regression analyses were conducted leaving out subjects that dropped out from the study (n=8).

Statistical significance was set at a *P* value <0.05. The Statistical Package for the Social Sciences (SPSS), version 28, was used for the analyses (SPSS Inc., Chicago, IL, U.S.A.).

Results

Seventy-nine patients with a mean age of 63.9 (10.4) years, 29 women, mean BMI of 27.5 (4.5) kg/m^2 were enrolled and allocated to either SMS intervention (n =40) or control group

For peer review only

For peer review only - http://bmjopen.bmj.com/site/about/guidelines.xhtml

(*n* =39) (13). Assessments were performed with a median of five (IQR 6) days after stroke or TIA and after three months. At baseline assessments, seven individuals temporarily used a walking aid. A majority of the included individuals did not receive rehabilitation until the measurement point of three months. Seventy-one individuals remained in the study at three months and eight individuals had dropped out.

Table 1 gives the clinical characteristics for all individuals at baseline and changes in the 6-minute walk test.

---*Insert Table 1 about here*---

At baseline, 27% of participants had a BMI ≥30 (obesity), 43% had a BMI between 25 and 29.9 (overweight), and 30% had a BMI <25. In this study, all participants could perform the 6-minute walk test on both occasions and no adverse advents occurred during testing. At baseline, the median (IQR) 6-minute walk test was 478 (141) meters. At three months, the median 6-minute walk test was 538 (158) meters. The median (IQR) change in the 6-minute walk test after three months was 57 (63) and 23 (73) meters for the SMS and control groups, respectively (P =0.037) (13). On average, the participants in the SMS group walked 6335 steps per day in the first week of intervention and 8173 steps per day after three months (P<0.001), an increase of 29% (n =33).

Linear regression analyses for the identification of factors related to changes in walking capacity

Table 2 shows correlations of possible variables for the regression models.

---*Insert Table 2 about here*---

For peer review only - http://bmjopen.bmj.com/site/about/guidelines.xhtml

The differences in walking performance were significantly associated in a univariate analysis with baseline BMI and the 6-minute walk test at baseline (Table 3). After adjusting for age, sex, and comorbidity, the final model still included baseline 6MWT and BMI, which together with age, sex, and no comorbidity explained 36% of the variance (Table 3). Younger individuals, men, and those with no comorbidity, lower baseline BMI, and shorter 6-minute walk test at baseline were more likely to improve their walking performance.

---Insert Table 3 about here---

The sensitivity analyses showed that BMI was no longer a strong predictor for changes in the 6-minute walk test in the complete case analyses (n=71). Thus, complete case analyses are presented in Table 4.

---Insert Table 4 about here---

Discussion

In this post hoc study, we showed in regression analyses that younger age, male sex, no comorbidity, lower baseline BMI, and less distance walked in 6-minute walk test significantly predicted positive change in 6-minute walk test three months after stroke or TIA.

In our study sample, those with higher age improved less in the 6-minute walk test at three months. Thus, our finding is in line with a general tendency to be less physically active at older ages (14). Age-related physiological changes like reduced oxygen uptake capacity (VO_2 max), changes in heart and lungs, loss of muscle mass and strength, more comorbidity, and

For peer review only - http://bmjopen.bmj.com/site/about/guidelines.xhtml

medications in older age may affect the intensity and ability to perform outdoor walking in the present study (1, 23). However, each individual in this study could find their own suggested intensity level by using the Borg scale.

In the present study, about 70% of the participants had a BMI above 25. Higher BMI predicted less improvement in walking distance as measured with the 6-minute walk test. It can be speculated that those with obesity also were sarcopenic; i.e., displayed sarcopenic obesity, which is known to affect walking performance (23). In our study sample, we found weight fluctuation in both directions after three months, which might have affected the results (22). A deterioration in health might be seen in individuals with high BMI due to difficulties being active in daily living. This study indicates that individuals after a stroke and with obesity need help initiating lifestyle changes to increase physical activity.

In a longitudinal study of cardiovascular disease secondary prevention, an inverse association between walking speed and mortality was found; with a 53% reduction in mortality risk for those with the highest walking speed (3.8–6.2 km/h) (24). In contrast to the present study, the participants were all women, but they had a similar risk factor profile (24). Another cross-sectional study, using a population three months or longer after stroke found that balance as measured with the Berg Balance Scale was a significant predictor of free-living walking activity, and explained 13% of the variance (12). In our selected sample of individuals with high motor functions at baseline, balance was not a major problem (13).

Fini et al. reported a mean of 4078 steps per day, six months or more after stroke. (25) The number of steps we found after the first week of SMS intervention in our sample with recent stroke and TIA is higher than that, averaging 6335 steps per day the first week of intervention and a further increase of 29% after three months. In older adults, a dose–response relationship

For peer review only - http://bmjopen.bmj.com/site/about/guidelines.xhtml

For peer review only

has been observed for sedentary behavior, as well as between steps per day and mortality (26). Walking speed, steps, and distance can reflect functional status and health and are important for activities and community ambulation in daily life (21). Furthermore, this study sample included more males which could have an impact on the results since male sex was associated with greater improvement in walking performance. In contrast to this study with individuals walking independently at study start, gender did not affect the outcome of a larger study (AVERT trial) examining factors associated with walking recovery post-stroke (27).

The reason why those who walked shorter distances at baseline improved the most is unknown, but regression toward the mean cannot be excluded. Still, in those with the poorest walking performance, a smaller increase in steps may be sufficient to have a positive impact on health (28). New epidemiological studies measuring physical activity with accelerometers show that the positive effects of physical activity may have been underestimated (28). A large prospective study indicated that up to 10,000 steps a day were associated with a lower risk of cardiovascular incidence and mortality (28). Additional risk reduction was also found with steps performed at a higher intensity and there was no minimum threshold for the association between increasing steps per day with morbidity and mortality (28). This can be used to motivate the least active individuals to increase their outdoor walking and number of steps per day.

Limitations and strengths

Some methodological issues need to be addressed in this study. One limitation is that the study design calls for precaution with causal inferences. Another limitation is that our findings cannot be generalized to more disabled community-living individuals after a stroke

For peer review only - http://bmjopen.bmj.com/site/about/guidelines.xhtml

or to individuals with chronic stroke. However, since we included participants at the hospital with different socio-economic statuses and educational backgrounds, we believe this sample to be representative of the acute stroke and TIA population with fewer motor deficits. Finally, the study is relatively small, making the study prone to bias, and all patients are from a single center in Sweden, therefore reducing the generalizability of the results.

One strength of the present study is that study data were drawn from a randomized controlled trial and that we used established outcome measures.

Conclusion: In summary, younger age, male sex, no comorbidity, lower BMI, and shorter 6-minute walk test at baseline contributed most to improvement in walking performance in patients with a recent stroke or TIA. These factors may be important when planning secondary prevention actions. Cost-effective and easily delivered interventions for individuals with minor stroke or TIA to increase walking distance still require further targeted research.

Author affiliations:

Birgit Vahlberg, Ph.D., RPT Department of Public Health and Caring Sciences, Geriatrics, Uppsala University, Uppsala, Sweden.

Staffan Eriksson, Ph.D., RPT Department of Public Health and Caring Sciences, Geriatrics, Uppsala University, Uppsala, and Centre for Clinical Research, Sörmland, Uppsala University, Eskilstuna, Sweden.

Ulf Holmbäck, Associate Professor, Department of Public Health and Caring Sciences, Clinical Nutrition and Metabolism, Uppsala University, Uppsala, Sweden.

For peer review only - http://bmjopen.bmj.com/site/about/guidelines.xhtml

Erik Lundström, Professor, MD, Department of Medical Sciences, Neurology, Uppsala University, Sweden.

Contributors: This study was conceived, organized, and managed by BV, SE, UH, and EL. BV acts as a guarantor of the study. All authors listed above contributed to the study design and data interpretation. Writing of the first draft of the paper was done by BMV and all authors were involved in the preparation and critique of the manuscript and reviewed the paper before submission.

Funding: This study was funded by the Medical Faculty at Uppsala University (grant number N/A), the Swedish National Stroke Association (Strokeförbundet) (grant number N/A), and the Geriatric Funding, Sweden (grant number N/A).

Patient consent for publication: Not applicable.

Competing interests: The author(s) report that there are no competing interests to declare.

Ethics approval: This study involves human participants and was approved by the regional Ethical Review Board of Uppsala University Hospital, Sweden: Dnr: 2015/550.

Data availability statement: The data set associated with this work is available from the corresponding author upon reasonable request.

For peer review only - http://bmjopen.bmj.com/site/about/guidelines.xhtml

ORCiD iDs

BV: http://orcid.org/0000-0002-1508-1435

SE: http://orcid.org/0000-0003-3572-8918

UH: http://orcid.org/0000-0002-0764-5878

EL: http://orcid.org/0000-0002-5313-9052

Figure: None.

References

1. Virani SS, Alonso A, Aparicio HJ, Benjamin EJ, Bittencourt MS, Callaway CW, et al. Heart Disease and Stroke Statistics-2021 Update: A Report From the American Heart Association. Circulation. 2021;143(8):e254-e743.
2. RIKSstroke. Stroke och TIA. Riksstrokes årsrapport från 2019 års data. Umeå; Riksstroke 2020. available from:htpp://www.riksstroke.org/wp-content/uploads/2020/09/Riksstroke_Årsrapport-2019_slutversionWEB-1.pdf; (cited 15 May, 2021); .
3. English C, Manns PJ, Tucak C, Bernhardt J. Physical activity and sedentary behaviors in people with stroke living in the community: a systematic review. Phys Ther. 2014;94(2):185-96.
4. Tudor-Locke C, Bassett DR. How many steps/day are enough? Preliminary pedometer indices for public health. Sports Med. 2004;34(1):1-8.
5. Saunders DH, Sanderson M, Hayes S, Kilrane M, Greig CA, Brazzelli M, et al. Physical fitness training for stroke patients. Cochrane Database Syst Rev. 2016;3:CD003316.
6. Field N, Gebruers N, Sundaram TS, Nicholson S, Mead G. Physical activity after stroke: A systematic review and meta-analysis. ISRN Stroke. 2013.;1.
7. Fulk GD, He Y, Boyne P, Dunning K. Predicting Home and Community Walking Activity Poststroke. Stroke. 2017;48(2):406-11.
8. Saunders DH, Sanderson M, Hayes S, Johnson L, Kramer S, Carter DD, et al. Physical fitness training for stroke patients. Cochrane Database Syst Rev. 2020;3(3): CD003316.
9. O'Donnell MJ, Chin SL, Rangarajan S, Xavier D, Liu L, Zhang H, et al. Global and regional effects of potentially modifiable risk factors associated with acute stroke in 32 countries (INTERSTROKE): a case-control study. Lancet. 2016;388(10046):761-75.

For peer review only - http://bmjopen.bmj.com/site/about/guidelines.xhtml

10. McKee M, Leon DA, Tomkins S, Shkolnikov VM, Andreev E. INTERHEART. Lancet. 2005;365(9454):117-8; author reply 8.
11. Tsao CW, Aday AW, Almarzooq ZI, Anderson CAM, Arora P, Avery CL, et al. Heart Disease and Stroke Statistics-2023 Update: A Report From the American Heart Association. Circulation. 2023;147(8):e93-e621.
12. van de Port I, Punt M, Meijer JW. Walking activity and its determinants in free-living ambulatory people in a chronic phase after stroke: a cross-sectional study. Disabil Rehabil. 2020;42(5):636-41.
13. Vahlberg B, Lundström E, Eriksson S, Holmbäck U, Cederholm T. Effects on walking performance and lower body strength by short message service guided training after stroke or transient ischemic attack (The STROKEWALK Study): a randomized controlled trial. Clin Rehabil. 2021;35(2):276-87.
14. Cuschieri S. The STROBE guidelines. Saudi J Anaesth. 2019;13(Suppl 1):S31-S4.
15. Brooks D, Solway S, Gibbons W. ATS statement on six-minute walk test. Am J Respir Crit Care Med. 2003;167(9):1287.
16. Banks JL, Marotta CA. Outcomes validity and reliability of the modified Rankin scale: implications for stroke clinical trials: a literature review and synthesis. Stroke. 2007;38(3):1091-6.
17. Nasreddine ZS, Phillips NA, Bédirian V, Charbonneau S, Whitehead V, Collin I, et al. The Montreal Cognitive Assessment, MoCA: a brief screening tool for mild cognitive impairment. J Am Geriatr Soc. 2005;53(4):695-9.
18. Goldstein LB, Samsa GP, Matchar DB, Horner RD. Charlson Index comorbidity adjustment for ischemic stroke outcome studies. Stroke. 2004;35(8):1941-5.
19. Guralnik JM, Simonsick EM, Ferrucci L, Glynn RJ, Berkman LF, Blazer DG, et al. A short physical performance battery assessing lower extremity function: association with self-reported disability and prediction of mortality and nursing home admission. J Gerontol. 1994;49(2):M85-94.
20. Guralnik JM, Ferrucci L, Simonsick EM, Salive ME, Wallace RB. Lower-extremity function in persons over the age of 70 years as a predictor of subsequent disability. N Engl J Med. 1995;332(9):556-61.
21. Studenski S, Perera S, Patel K, Rosano C, Faulkner K, Inzitari M, et al. Gait speed and survival in older adults. JAMA. 2011;305(1):50-8.
22. Vahlberg BM, Lundström E, Eriksson S, Holmback U, Cederholm T. Potential effects on cardiometabolic risk factors and body composition by short message service (SMS)-guided training after recent minor stroke or transient ischaemic attack: post hoc analyses of the STROKEWALK randomised controlled trial. BMJ Open. 2021;11(10):e054851.
23. Cruz-Jentoft AJ, Bahat G, Bauer J, Boirie Y, Bruyère O, Cederholm T, et al. Sarcopenia: revised European consensus on definition and diagnosis. Age Ageing. 2019;48(4):601.
24. Raisi A, Zerbini V, Myers J, Masotti S, Piva T, Lordi R, et al. Moderate walking speed and survival association across 23-years follow-up in female patients with cardiovascular disease. Int J Cardiol. 2023;371:371-6.
25. Fini NA, Holland AE, Keating J, Simek J, Bernhardt J. How Physically Active Are People Following Stroke? Systematic Review and Quantitative Synthesis. Phys Ther. 2017;97(7):707-17.
26. Rojer AGM, Ramsey KA, Trappenburg MC, van Rijssen NM, Otten RHJ, Heymans MW, et al. Instrumented measures of sedentary behaviour and physical activity are

For peer review only

For peer review only - http://bmjopen.bmj.com/site/about/guidelines.xhtml

associated with mortality in community-dwelling older adults: A systematic review, meta-analysis and meta-regression analysis. Ageing Res Rev. 2020;61:101061.

27. Kennedy C, Bernhardt J, Churilov L, Collier JM, Ellery F, Rethnam V, et al. Factors associated with time to independent walking recovery post-stroke. J Neurol Neurosurg Psychiatry. 2021;92(7):702-8.

28. Del Pozo Cruz B, Ahmadi MN, Lee IM, Stamatakis E. Prospective Associations of Daily Step Counts and Intensity With Cancer and Cardiovascular Disease Incidence and Mortality and All-Cause Mortality. JAMA Intern Med. 2022;182(11):1139-48.

For peer review only

Table 1. Baseline characteristics of all patients in the study with ≥34 meters increase in the six-minute walking test at three months versus patients with < 34 meters increase in the six-minute walking test. The Student´s t-test was used for continuous, normally distributed variables, and the Mann Whitney-U test was applied for ordinal or non-normally distributed variables. The Chi-square test was used for categorical variables.

For peer review only

	**Study population**	**Changes in the 6-minute walking test**			
	**Baseline**	**≥ 34 m (n=38)**	**<34 m (n=41)**	**P-value**	**Missing values,** n (%)
Age, mean (SD)	63.9 (10.4)	61.9 (9.1)	65.7 (11.2)	0.01	0
Female, n (%)	29 (36.7)	11 (28.9)	18 (43.9)	0.17	0
SMS group, n (%)	40 (50.6)	26 (65.0)	14 (35.0)	0.002	0
Control group, n (%)	39 (49.4)	12 (30.8)	27 (69.2)		
modified Rankin Scale, 0-2				0.47	0
0	11 (13.9)	6 (54.5)	5 (45.5)		
1	53 (67.1)	23 (43.4)	30 (56.6)		
2	15 (19.0)	9 (60.0)	6 (40.0)		
Diagnosis, n (%)				0.70	0
Cerebral infarction	66 (83.5)	29 (50.9)	28 (49.1)		
Intracerebral hemorrhage	9 (11.4)	4 (44.4)	5(55.6)		
TIA	13 (16.5)	8 (61.5)	5 (38.5)		

For peer review only - http://bmjopen.bmj.com/site/about/guidelines.xhtml

Charlson Comorbidity Index, n (%)					0
No comorbidity	47 (59.5)	25 (53.2)	22 (46.8)	0.27	
≥1	32 (40.5)	13 (40.6)	19 (59.4)		
BMI, mean (SD)	27.5 (4.5)	26.55 (3.77)	28.39 (5.03)	0.14	0
Diabetes mellitus-2, yes n (%)	12 (15.2)	5 (13.2)	7 (17.1)	0.63	0
University studies, yes n (%)	40 (62)	16 (42.1)	24 (58.5)	0.14	0
Non-smoking, n (%)	71 (90)	33 (86.8)	38 (92.7)	0.39	0
Step counts (SMS group), mean (SD)	6335 (2747)	6612 (2741)	5757 (2786)	0.61	3 (7.5)
SGPALS, n (%)				0.47	0
Sedentary	11 (13.9)	6 (54.5)	5 (45.5)		
Light Physical Activity	53 (67.1)	23 (43.4)	30 (56.6)		
Moderate/High Physical Activity	15 (19.0)	9 (60.0)	6 (40.0)		
P-HDL cholesterol, (mmol/l), mean (SD)	1.32 (0.39)	1.35 (0.45)	1.26 (0.32)	0.44	3 (3.8)
P-LDL cholesterol, (mmol/l), mean (SD)	3.16 (1.09)	3.18 (1.16)	3.13 (1.04)	0.86	3 (3.8)
P-Cholesterol, (mmol/l), mean (SD)	5.13 (1.20)	5.16 (1.30)	5.13 (1.12)	0.86	3 (3.8)
P-Triglycerides, (mmol/l), mean (SD)	1.35 (0.63)	1.20 (0.46)	1.50 (0.74)	0.03	3 (3.8)
P-HbA1C, mmol/mol), mean (SD)	38.0 (7.54)	37.73 (8.33)	38.26 (6.77)	0.47	3 (3.8)
P-C reactive protein (mg/L), mean (SD)	4.92 (19.40)	6.80 (27.80)	3.20 (3.95)	0.20	6 (7.6)
P-Creatinine (mmol/L), mean (SD)	85.12 (25.53)	90.80	78.97 (17.83)	0.11	2 (2.6)
SBP, (mm HG), mean (SD)	130.63(16.56)	129.80 (16.17)	131.36 (17.09)	0.81	4 (5)
DBP, (mm HG), mean (SD)	76.08 (11.0)	79.17 (11.24)	73.23 (10.09)	0.02	4 (5)

Abbreviations: BMI, body mass index, CRP, C-reactive protein; DBP, diastolic blood pressure; HbA1C, glycated hemoglobin; HDL, high-density lipoprotein; LDL, low-density

For peer review only - http://bmjopen.bmj.com/site/about/guidelines.xhtml

483 lipoprotein; SGPALS, Saltin Grimby Physical Activity Scale; SMS, short message service
484 group; SBP, systolic blood pressure

485 **Table 2.** Correlation of the variables used in the regression models and changes in walking
486 performance. Correlation strength was calculated using Spearman´s rho for non-parametric
487 data or Pearson correlation for continuous normally distributed variables.

	Change 6-minute walking test, *r*	P-value
Age (years)	-0.39	<0.001
Sex (female)	-0.13	0.26
CCI (≥1 comorbidity)	-0.20	0.08
BMI, (kg/m^2)	-0.23	0.046
6-minute walking test, baseline (meters)	-0.38	<0.001
Chair-stand test, (seconds)	0.19	0.086
Montreal Cognitive Assessment scale, (≥26 points),	0.014	0.91
Saltin Grimby Physical Activity Level Scale (Sedentary, light physical activity, moderate/high physical activity)	0.078	0.49

488 Abbreviations: BMI, Body Mass Index; CCI, Charlson Comorbidity Index

489

For peer review only - http://bmjopen.bmj.com/site/about/guidelines.xhtml

For peer review only

For peer review only - http://bmjopen.bmj.com/site/about/guidelines.xhtml

Table 3. Univariate and multivariate regression analyses with changes in walking performance (6-minute walking test) as the dependent variable and age, sex, comorbidity, baseline BMI-, and baseline 6-minute walk test as independent variables in individuals after stroke and TIA. An intention-to-treat analysis was performed with drop-outs included (n=8).

	**Univariate analysis**				**Multivariate analysis**		
	**Beta standardized**	**Adjusted R square**	**95% CI**	**P**	**Beta standardized**	**95% CI**	**P**
Age, yrs	-20	0.029	-3.1 to 0.1	0.073	-0.33	-3.8 to -1.0	<0.001
Sex, female	-0.12	0.002	-54.4 to 16.4	0.29	-0.24	-66.9 to -8.0	0.014
CCI, ≥1 comorbidity	-0.19	0.024	-63.8 to 4.9	0.09	-0.16	-55.5 to 5.4	0.12
BMI, kg/m^2	-0.23	0.038	-7.5 to -0.07	0.046	-0.29	-8.1 to -1.6	0.004
6-minute walk test, meters	-0.38	0.134	-0.4 to -0.1	<0.001	-0.55	-0.5 to -0.3	<0.001

Abbreviations: BMI, Body Mass Index; CCI, Charlson Comorbidity Index

The adjusted R square for the multivariate analysis is 0.36.

For peer review only

500 **Table 4.** Sensitivity analysis: Univariate and multivariate regression analyses with changes in walking performance (6-minute walking test) as
501 the dependent variable and age, sex, comorbidity, baseline BMI-, and baseline 6-minute walk test as independent variables in individuals after
502 stroke and TIA. In the sensitivity analysis, individuals with missing data at three months were not analyzed (n=8)

	**Univariate analysis**				**Multivariate analysis**		
	**Beta standardized**	**Adjusted R square**	**95% CI**	**P**	**Beta standardized**	**95% CI**	**P**
Age, yrs	-0.25	0.05	-3.9 to -0.2	0.03	-0.33	-4.3 to -1.1	0.002
Sex, female	-0.11	-0.002	-57.5 to 20.8	0.35	-0.24	-72.7 to -7.1	0.018
CCI, ≥1 comorbidity	-0.18	0.018	-66.6 to 9.7	0.14	-0.22	-67.4 to -2.6	0.035
BMI, kg/m^2	-0.21	0.031	-8.1 to 0.43	0.08			
6-minute walk test, meters	-0.42	0.16	-0.47 to -0.15	<0.001	-0.55	-0.56 to -0.26	<0.001

503 Abbreviations: BMI, Body Mass Index; CCI, Charlson Comorbidity Index
504 The adjusted R square for the multivariate analysis is 0.34.
505

For peer review only - http://bmjopen.bmj.com/site/about/guidelines.xhtml

CONSORT CHECKLIST

Table. CONSORT 2010 Checklist of Information to Include When Reporting a Randomized Trial[a]

Section and Topic	Item No.	Checklist Item	Reported on Page No.
**Title and abstract**			
	1a	Identification as a randomized trial in the title	title pag
	1b	Structured summary of trial design, methods, results, and conclusions (for specific guidance see CONSORT for abstracts)	abstract
**Introduction**			
Background and objectives	2a	Scientific background and explanation of rationale	1-2
	2b	Specific objectives or hypotheses	1-2
**Methods**			
Trial design	3a	Description of trial design (such as parallel, factorial) including allocation ratio	2
	3b	Important changes to methods after trial commencement (such as eligibility criteria), with reasons	ref 13
Participants	4a	Eligibility criteria for participants	2
	4b	Settings and locations where the data were collected	2
Interventions	5	The interventions for each group with sufficient details to allow replication, including how and when they were actually administered	4-5
Outcomes	6a	Completely defined prespecified primary and secondary outcome measures, including how and when they were assessed	3-4
	6b	Any changes to trial outcomes after the trial commenced, with reasons	N/A
Sample size	7a	How sample size was determined	5
	7b	When applicable, explanation of any interim analyses and stopping guidelines	N/A
Randomization			
Sequence generation	8a	Method used to generate the random allocation sequence	ref 13
	8b	Type of randomization; details of any restriction (such as blocking and block size)	ref 13
Allocation concealment mechanism	9	Mechanism used to implement the random allocation sequence (such as sequentially numbered containers), describing any steps taken to conceal the sequence until interventions were assigned	ref 13
Implementation	10	Who generated the random allocation sequence, who enrolled participants, and who assigned participants to interventions	ref 13
Blinding	11a	If done, who was blinded after assignment to interventions (for example, participants, care providers, those assessing outcomes) and how	ref 13
	11b	If relevant, description of the similarity of interventions	N/A
Statistical methods	12a	Statistical methods used to compare groups for primary and secondary outcomes	5
	12b	Methods for additional analyses, such as subgroup analyses and adjusted analyses	6
**Results**			
Participant flow (a diagram is strongly recommended)	13a	For each group, the numbers of participants who were randomly assigned, received intended treatment, and were analyzed for the primary outcome	ref 13
	13b	For each group, losses and exclusions after randomization, together with reasons	ref 13
Recruitment	14a	Dates defining the periods of recruitment and follow-up	ref 13, F
	14b	Why the trial ended or was stopped	N/A
Baseline data	15	A table showing baseline demographic and clinical characteristics for each group	Table 1
Numbers analyzed	16	For each group, number of participants (denominator) included in each analysis and whether the analysis was by original assigned groups	ref 13
Outcomes and estimation	17a	For each primary and secondary outcome, results for each group, and the estimated effect size and its precision (such as 95% confidence interval)	ref 13
	17b	For binary outcomes, presentation of both absolute and relative effect sizes is recommended	N/A
Ancillary analyses	18	Results of any other analyses performed, including subgroup analyses and adjusted analyses, distinguishing prespecified from exploratory	T 4
Harms	19	All important harms or unintended effects in each group (for specific guidance see CONSORT for harms)	ref 11
**Comment**			
Limitations	20	Trial limitations, addressing sources of potential bias, imprecision, and, if relevant, multiplicity of analyses	below al
Generalizability	21	Generalizability (external validity, applicability) of the trial findings	10-11
Interpretation	22	Interpretation consistent with results, balancing benefits and harms, and considering other relevant evidence	ref 13
**Other information**			
Registration	23	Registration number and name of trial registry	abstract
Protocol	24	Where the full trial protocol can be accessed, if available	pilot stu
Funding	25	Sources of funding and other support (such as supply of drugs), role of funders	12

[a] We strongly recommend reading this statement in conjunction with the CONSORT 2010 Explanation and Elaboration for important clarifications on all the items. If relevant, we also recommend reading CONSORT extensions for cluster randomized trials, noninferiority and equivalence trials, nonpharmacological treatments, herbal interventions, and pragmatic trials. Additional extensions are forthcoming: for those and for up-to-date references relevant to this checklist, see http://www.consort-statement.org.

©2010 American Medical Association. All rights reserved.

For peer review only

For peer review only - http://bmjopen.bmj.com/site/about/guidelines.xhtml

Factors associated with changes in walking performance in individuals three months after stroke or TIA—secondary analyses from a randomized controlled trial of SMS-delivered training instructions in Sweden

Journal:	*BMJ Open*
Manuscript ID	bmjopen-2023-078180.R2
Article Type:	Original research
Date Submitted by the Author:	03-Feb-2024
Complete List of Authors:	Vahlberg, Birgit; Uppsala University, Department of Public Health and Caring Sciences, Geriatrics<br>Eriksson, Staffan; Uppsala University, Department of Public Health and Caring Sciences, Uppsala University; Uppsala University, Centre for Clinical Research, Sörmland, Eskilstuna;<br>Holmbäck, Ulf; Uppsala University, Department of Public Health and Caring Sciences, Clinical Nutrition and Metabolism,<br>Lundström, Erik; Uppsala University, Department of Medical sciences, Neurology
**Primary Subject Heading**:	Rehabilitation medicine
Secondary Subject Heading:	Neurology, Geriatric medicine, Public health
Keywords:	Stroke < NEUROLOGY, Risk Factors, REHABILITATION MEDICINE, Clinical Trial

SCHOLARONE™
Manuscripts

I, the Submitting Author has the right to grant and does grant on behalf of all authors of the Work (as defined in the below author licence), an exclusive licence and/or a non-exclusive licence for contributions from authors who are: i) UK Crown employees; ii) where BMJ has agreed a CC-BY licence shall apply, and/or iii) in accordance with the terms applicable for US Federal Government officers or employees acting as part of their official duties; on a worldwide, perpetual, irrevocable, royalty-free basis to BMJ Publishing Group Ltd ("BMJ") its licensees and where the relevant Journal is co-owned by BMJ to the co-owners of the Journal, to publish the Work in this journal and any other BMJ products and to exploit all rights, as set out in our licence.

The Submitting Author accepts and understands that any supply made under these terms is made by BMJ to the Submitting Author unless you are acting as an employee on behalf of your employer or a postgraduate student of an affiliated institution which is paying any applicable article publishing charge ("APC") for Open Access articles. Where the Submitting Author wishes to make the Work available on an Open Access basis (and intends to pay the relevant APC), the terms of reuse of such Open Access shall be governed by a Creative Commons licence – details of these licences and which Creative Commons licence will apply to this Work are set out in our licence referred to above.

Other than as permitted in any relevant BMJ Author's Self Archiving Policies, I confirm this Work has not been accepted for publication elsewhere, is not being considered for publication elsewhere and does not duplicate material already published. I confirm all authors consent to publication of this Work and authorise the granting of this licence.

For peer review only

Factors associated with changes in walking performance in individuals three months after stroke or TIA—secondary analyses from a randomized controlled trial of SMS-delivered training instructions in Sweden

Short title: Predicting changes in walking performance three months after recent stroke and TIA in Sweden.

Birgit Vahlberg, PhD, RPT, Department of Public Health and Caring Sciences, Geriatrics, Uppsala University, Uppsala.

Staffan Eriksson, PhD, RPT, Department of Public Health and Caring Sciences, Geriatrics, Uppsala University, Uppsala; Centre for Clinical Research, Sörmland, Uppsala University, Eskilstuna.

Ulf Holmbäck, Associate Professor, Department of Public Health and Caring Sciences, Clinical Nutrition and Metabolism, Uppsala University, Uppsala.

Erik Lundström, Professor, MD, Department of Medical Sciences, Neurology, Uppsala University, Uppsala.

All authors are from Sweden.

Corresponding author: Birgit Vahlberg

Address: Department of Public Health and Caring Sciences, Geriatrics,

Uppsala University, BMC A9

S-751 24 Uppsala, Sweden

Telephone: +46 709583473

E-mail: Birgit.Vahlberg@pubcare.uu.se

Word count main text: 3133

Keywords: stroke, risk factors, rehabilitation medicine, clinical trial

For peer review only

ABSTRACT:

Objectives: To identify factors related to changes in walking performance in individuals 3 months after a stroke or TIA.

Design: Cross-sectional study with post-hoc analysis of a randomized controlled study.

Setting: University Hospital, Sweden.

Participants: 79 individuals, 64 (10) years, 37% women, who were acutely hospitalized because of stroke or TIA between November 2016 and December 2018. Inclusion criteria were patients aged 18 or above and the major eligibility criterion was the ability to perform the 6-minute walking test.

Intervention: The intervention group received standard care plus daily mobile phone text messages (SMS) with instructions to perform regular outdoor walking and functional leg exercises in combination with step counting and training diaries. The control group received standard care.

Outcome measures: Multivariate analysis was performed and age, sex, group allocation, comorbidity, baseline 6-minute walk test, BMI, cognition, and chair-stand tests were entered as possible determinants for changes in the 6-minute walk test.

Results: Multiple regression analyses showed that age (Standardized Beta -0.33, 95% CI -3.8 to -1.05, *P*<0.001), sex (-0.24, 95% CI -66.9 to -8.0, *P* = 0.014), no comorbidity (-0.16, 95% CI -55.5 to 5.4, *P* = 0.11), baseline BMI (-0.29, 95% CI -8.1 to -1.6, *P* = 0.004), baseline 6-minute walk test (-0.55, 95% CI -0.5 to -0.3, *P* <0.001) were associated with changes in 6-minute walk test three months after the stroke event. The regression model described 36% of the variance in changes in the 6-minute walk test.

For peer review only

Conclusions: Post-hoc regression analyses indicated that younger age, male sex, lower BMI, and shorter 6-minute walk test at baseline and possible no comorbidity contributed to improvement in walking performance at three months in patients with a recent stroke or TIA. These factors may be important when planning secondary prevention actions.

Clinical Trial Registry: ClinicalTrial.gov, number NCT02902367

STRENGTHS AND LIMITATIONS OF THIS STUDY

Study data were drawn from a randomized controlled trial and we used established outcome measures.

The study design calls for precaution with causal inferences.

Our findings cannot be generalized to more disabled community-living individuals after a stroke or to individuals with chronic stroke.

The study is relatively small, making the study prone to bias, and all patients are from a single center in Sweden, reducing the generalizability of the results.

For peer review only

Introduction

Approximately three out of four stroke incidents can be attributed to behavioral risk factors—for example, unhealthy diets and low levels of physical activity (1, 2). Both stroke and TIA indicate ongoing arteriosclerotic changes in the vessels that can lead to further cardiovascular events (1, 2). Physical activity is known to decrease the risk of stroke, TIA, and myocardial infarction (3-5).

After a stroke, individuals are often predisposed to functional limitations, contributing to a further risk of recurrent stroke or cardiovascular complications (1, 6). Individuals who have suffered from a stroke take an average of 4000 steps a day in the chronic phase after stroke, which is far below the recommended 10,000 steps to meet the guidelines for physical activity (6, 7). Also, individuals with high function after a stroke limit their activity. Still, we know that even low-intensity physical activity, such as walking, is important for minimizing disability and promoting long-term metabolic health (8, 9). Outdoor walking is generally easy to perform. It is a cost-effective measure to address sedentary lifestyles and increase physical activity after stroke.

Research demonstrates that the ability to walk longer distances and good balance correlates with higher physical activity levels following a stroke (9, 10). Thus, interventions for enhancing walking performance are of crucial importance for secondary prevention (11). Metrics such as walking distance can be utilized as an indicator of the level of physical activity after a stroke or TIA. In addition, understanding factors associated with improved walking is also essential for developing targeted interventions.

For peer review only - http://bmjopen.bmj.com/site/about/guidelines.xhtml

There is a lack of studies investigating changes in walking performance shortly following a stroke and TIA (11, 12). In the STROKEWALK study, a previously-reported randomized controlled trial, we found that individuals receiving text messages for three months after stroke and TIA coupled with add-on interventions using a training diary and step counts improved walking distance and chair-rising performance (13). The present study aims to further study how various baseline characteristics including cardio-metabolic risk markers relate to changes in the 6-minute walk test, using secondary analyses of data from the STROKEWALK study.

Methods

Study design

This is a post-hoc analysis of the STROKEWALK randomized controlled study performed at the Uppsala University Hospital in Sweden. Determination of sample size, recruitment, data collection, and random allocation procedures have been previously described in detail (13). Ethical approval was obtained from the regional Ethical Review Board of Uppsala University Hospital, Sweden: Dnr: 2015/550. The present study report follows the Strengthening the Reporting of Observational Studies in Epidemiology (STROBE) guidelines for reporting cross-sectional studies (14) and written consent was obtained from all participants.

Recruitment was initiated in November 2016, and the last three-month follow-up assessment was performed in December 2018. Included were participants aged 18 or above with TIA or verified stroke (infarction or intracerebral hemorrhage with first or recurrent event) with sufficient cognition (Montreal Cognitive Assessment scale, MoCA ≥26 points), general disability (modified Rankin Scale ≥2), and good enough walking performance; i.e., ability to

For peer review only - http://bmjopen.bmj.com/site/about/guidelines.xhtml

perform the six-minute walking test (with or without a walking aid) (13). The exclusion criteria were known subarachnoid hemorrhage, medical problems such as uncontrolled hypertension, untreated arrhythmias, unstable cardiovascular conditions, a dementia diagnosis, severe aphasia, severe psychiatric problems, or cognitive impairment with difficulties understanding instructions (13).

Study outcomes

The 6-minute walk test was used to measure the maximal walking distance during six minutes over a 30-meter course. Changes are described as differences in walking distance at three months (13, 15). All baseline data were collected on one occasion while the patients were still treated at the hospital or the first days after discharge and after three months close to the end of interventions.

Baseline assessments

All baseline data were collected on one occasion while the patients were still treated at the hospital or the first days after discharge.

The modified Rankin scale was used to assess general disability and is scored from 0 (no symptoms) to 6 (dead) (13, 16).

Cognitive function at baseline was assessed using the Montreal Cognitive Assessment scale (0–30 points),(13, 17) with a higher value indicating better function.

The Charlson Comorbidity Index (CCI) was used to classify comorbid conditions (13, 18).

For peer review only - http://bmjopen.bmj.com/site/about/guidelines.xhtml

The last registration of supine blood pressure was recorded manually before discharge from the hospital. Smoking and education levels were assessed by yes or no answers to the questions: 'Are you a smoker at this time of your life?' and 'Do you have a university degree?' For the chair-stand test, the participant was instructed to rise from a seated position without support as quickly as possible five times in a row (13, 19). The test was performed with standardized instructions from the Short Physical Performance Battery (13, 20). The chair-stand test was a measure of lower body strength and the severity of the lower limb impairment.

The 10-meter walk test was used to measure comfortable walking speed (13, 21).

From the patient's medical records, cardio-metabolic biochemical risk factors such as total, LDL, and HDL cholesterol, C-reactive protein (CRP), and triglycerides were analyzed at hospital admission and diagnoses such as diabetes, hypertension, hypercholesterolemia, and cardiac heart failure were registered (22). Biochemical analyses were performed by accredited methods of the Clinical Chemistry Laboratory at the Uppsala University Hospital, Uppsala. Glycated hemoglobin (HbA1C) was analyzed using non-fasting venous blood samples.

Body mass index was calculated as body weight (kg) divided by height (m) squared. Weight was recorded with participants wearing light indoor clothing. Height was measured to the nearest cm. A hip-mounted pedometer at weeks 1 and 12 was used for daily step count by individuals in the SMS group (Yamax, SW-200).

The SMS intervention group

The SMS-intervention group received daily text SMS (no cost for the participants) as an addition to standard care with simple instructions on what and how to exercise for three months. The intervention in the SMS group was comprised of three different strategies: 1) three months of daily SMS-text messages, 2) training diaries, and 3) pedometers for step counts for the first and last week of intervention (13). The text messages gave instructions on how to exercise to increase walking endurance and strength of the lower body, without the possibility of texting back for help or advice.

The control group

Patients in the control group were given standard stroke unit care. They had no restrictions regarding physical activity, exercise, or taking part in rehabilitation services, and were given standard recommendations. The control group did not use pedometers since it was considered to be a part of the intervention.

The number of individuals that were taking part in rehabilitation services during the study was not recorded.

Patient and Public Involvement Statement: A previous pilot study was conducted to test the design of the randomized controlled trial. The intervention was designed in collaboration between individuals with stroke and TIA, healthcare professionals, and researchers.

Statistics

In the STROKEWALK study, a sample size of 80 individuals (including a possible drop-out rate of 20%) was determined to provide secure power to detect a 34-meter clinically relevant mean difference in the 6-minute walking test (13). An intention-to-treat analysis was applied for all missing values (dropouts), and the change was assumed to be zero. Thus, follow-up data for dropouts were registered with a baseline carry-forward approach. Descriptive data

For peer review only - http://bmjopen.bmj.com/site/about/guidelines.xhtml

are reported as means (SD) and medians (IQR). According to the histogram, normal Q–Q plots, and the Kolmogorov–Smirnov test, data on changes in the 6-minute walk test were normally distributed. Case-wise diagnostics and standardized residuals were used to identify potential outliers. Differences in step counts from baseline to three months for the SMS group were calculated with the Student´s paired-sample t-test. Baseline differences between those that improved ≥34 meters or <34 meters in the 6-minute walk test were assessed using the Student´s t-test for continuous, normally distributed variables, and the Mann Whitney-U test was applied for ordinal or non-normally distributed variables. The Chi-square test was used for categorical variables. The cut-off of 34 meters was used for power analyses in the original study (13).

In the regression analyses, baseline explanatory variables for changes in the 6-minute walk test were first identified by correlation and univariate regression analyses ($P < 0\ .05$). Correlation strength was calculated using Spearman´s rho for non-parametric data or a Pearson correlation for continuous normally distributed variables. The identified variables were checked for multi-collinearity by correlation analysis and cross-tabulation and if the correlation coefficient was 0.80 or more the variable with the lowest r in relation to the dependent variable was omitted from further regression analysis. The baseline 10-meter walk test was omitted from further analyses due to multi-collinearity. Multiple linear regression analyses were then conducted with the remaining variables to discover which had the greatest impact on changes in walking performance. The ordinal explanatory variable Charlson Comorbidity Index was dichotomized and grouped into “no comorbidity” or “one or more than one comorbidity”. The ordinal explanatory variable Montreal Cognitive Assessment scale was dichotomized and grouped to the cutoff score ≥26 points. Changes in the 6-minute walk test were used as the dependent variable. We adjusted for age, sex, and comorbidity. Case-wise diagnostics showed that one individual could be considered an outlier; i.e., with an

For peer review only - http://bmjopen.bmj.com/site/about/guidelines.xhtml

increase in the 6-minute walk test of 365 meters, but were not omitted from further analysis. In this study, a sensitivity analysis with complete case analyses was also carried out. The univariate and multivariate regression analyses were conducted leaving out subjects that dropped out from the study (n=8).

Statistical significance was set at a *P* value <0.05. The Statistical Package for the Social Sciences (SPSS), version 28, was used for the analyses (SPSS Inc., Chicago, IL, U.S.A.).

Results

Seventy-nine patients with a mean age of 63.9 (10.4) years, 29 women, mean BMI of 27.5 (4.5) kg/m^2 were enrolled and allocated to either SMS intervention (*n* =40) or control group (*n* =39) (13). Assessments were performed with a median of five (IQR 6) days after stroke or TIA and after three months. At baseline assessments, seven individuals temporarily used a walking aid. Seventy-one individuals remained in the study at three months and eight individuals had dropped out.

Table 1 gives the clinical characteristics for all individuals at baseline and changes in the 6-minute walk test.

---Insert Table 1 about here---

At baseline, 27% of participants had a BMI ≥30 (obesity), 43% had a BMI between 25 and 29.9 (overweight), and 30% had a BMI <25. In this study, all participants could perform the 6-minute walk test on both occasions and no adverse advents occurred during testing. At baseline, the median (IQR) 6-minute walk test was 478 (141) meters. At three months, the median 6-minute walk test was 538 (158) meters. The median (IQR) change in the 6-minute walk test after three months was 57 (63) and 23 (73) meters for the SMS and control groups,

For peer review only

For peer review only - http://bmjopen.bmj.com/site/about/guidelines.xhtml

respectively and the SMS group showed a significantly greater increase in walking distance compared to the control group (*P* =0.037) (13). On average, the participants in the SMS group walked 6335 steps per day in the first week of intervention and 8173 steps per day after three months (*P*<0.001), an increase of 22.5% (n =33).

Linear regression analyses for the identification of factors related to changes in walking capacity

Table 2 shows correlations of possible variables for the regression models.

---Insert Table 2 about here---

The differences in walking performance were significantly associated in univariate analysis with baseline BMI and the 6-minute walk test at baseline (Table 3). After adjusting for age, sex, and comorbidity, the final model still included a baseline 6-minute walk test and BMI, which together with age, sex, and no comorbidity explained 36% of the variance (Table 3). Younger individuals, men, and those with no comorbidity, lower baseline BMI, and shorter 6-minute walk tests at baseline were more likely to improve their walking performance.

---Insert Table 3 about here---

The sensitivity analyses showed that BMI was no longer a strong predictor for changes in the 6-minute walk test in the complete case analyses (n=71). Thus, complete case analyses are presented in Table 4.

For peer review only - http://bmjopen.bmj.com/site/about/guidelines.xhtml

---Insert Table 4 about here---

Discussion

In this post hoc study, we showed in regression analyses that younger age, male sex, no comorbidity, lower baseline BMI, and less distance walked in 6-minute walk test significantly predicted positive change in 6-minute walk test three months after stroke or TIA.

In our study sample, those with higher age improved less in the 6-minute walk test at three months. Thus, our finding is in line with a general tendency to be less physically active at older ages (14). Age-related physiological changes like reduced oxygen uptake capacity (VO_2 max), changes in heart and lungs, loss of muscle mass and strength, more comorbidity, and medications in older age may affect the intensity and ability to perform outdoor walking in the present study (1, 23). However, each individual in this study could find their own suggested intensity level by using the Borg scale.

In the present study, about 70% of the participants had a BMI above 25. Higher BMI predicted less improvement in walking distance as measured with the 6-minute walk test. It can be speculated that those with obesity also were sarcopenic; i.e., displayed sarcopenic obesity, which is known to affect walking performance (23). In our study sample, we found weight fluctuation in both directions after three months, which might have affected the results (22). A deterioration in health might be seen in individuals with high BMI due to difficulties being active in daily living. This study indicates that individuals after a stroke and with obesity need help initiating lifestyle changes to increase physical activity.

In a longitudinal study of cardiovascular disease secondary prevention, an inverse association between walking speed and mortality was found; with a 53% reduction in mortality risk for

For peer review only - http://bmjopen.bmj.com/site/about/guidelines.xhtml

For peer review only

those with the highest walking speed (3.8–6.2 km/h) (24). In contrast to the present study, the participants were all women, but they had a similar risk factor profile (24). Another cross-sectional study, using a population three months or longer after stroke found that balance as measured with the Berg Balance Scale was a significant predictor of free-living walking activity, and explained 13% of the variance (12). In our selected sample of individuals with high motor functions at baseline, balance was not a major problem (13).

Fini et al. reported a mean of 4078 steps per day, six months or more after stroke. (25) The number of steps we found after the first week of SMS intervention in our sample with recent stroke and TIA is higher than that, averaging 6335 steps per day the first week of intervention and a further increase of 29% after three months. In older adults, a dose–response relationship has been observed for sedentary behavior, as well as between steps per day and mortality (26). Walking speed, steps, and distance can reflect functional status and health and are important for activities and community ambulation in daily life (21). Furthermore, this study sample included more males which could have an impact on the results since male sex was associated with greater improvement in walking performance. In contrast to this study with individuals walking independently at the study start, gender did not affect the outcome of a larger study (AVERT trial) examining factors associated with walking recovery post-stroke (27).

The reason why those who walked shorter distances at baseline improved the most is unknown, but regression toward the mean cannot be excluded. Still, in those with the poorest walking performance, a smaller increase in steps may be sufficient to have a positive impact on health (28). New epidemiological studies measuring physical activity with accelerometers

For peer review only - http://bmjopen.bmj.com/site/about/guidelines.xhtml

show that the positive effects of physical activity may have been underestimated (28). A large prospective study indicated that up to 10,000 steps a day were associated with a lower risk of cardiovascular incidence and mortality (28). Additional risk reduction was also found with steps performed at a higher intensity and there was no minimum threshold for the association between increasing steps per day with morbidity and mortality (28). This can be used to motivate the least active individuals to increase their outdoor walking and number of steps per day.

Limitations and strengths

Some methodological issues need to be addressed in this study. One limitation is that the study design calls for precaution with causal inferences. Another limitation is that our findings cannot be generalized to more disabled community-living individuals after a stroke or to individuals with chronic stroke. However, since we included participants at the hospital with different socio-economic statuses and educational backgrounds, we believe this sample to be representative of the acute stroke and TIA population with fewer motor deficits. Finally, the study is relatively small, making the study prone to bias, and all patients are from a single center in Sweden, therefore reducing the generalizability of the results.

One strength of the present study is that study data were drawn from a randomized controlled trial and that we used established outcome measures.

Conclusion: In summary, younger age, male sex, no comorbidity, lower BMI, and shorter 6-minute walk test at baseline contributed most to improvement in walking performance in patients with a recent stroke or TIA. These factors may be important when planning secondary prevention actions. Cost-effective and easily delivered interventions for

For peer review only - http://bmjopen.bmj.com/site/about/guidelines.xhtml

individuals with minor stroke or TIA to increase walking distance still require further targeted research.

Author affiliations:

Birgit Vahlberg, Ph.D., RPT Department of Public Health and Caring Sciences, Geriatrics, Uppsala University, Uppsala, Sweden.

Staffan Eriksson, Ph.D., RPT Department of Public Health and Caring Sciences, Geriatrics, Uppsala University, Uppsala, and Centre for Clinical Research, Sörmland, Uppsala University, Eskilstuna, Sweden.

Ulf Holmbäck, Associate Professor, Department of Public Health and Caring Sciences, Clinical Nutrition and Metabolism, Uppsala University, Uppsala, Sweden.

Erik Lundström, Professor, MD, Department of Medical Sciences, Neurology, Uppsala University, Sweden.

Contributors: This study was conceived, organized, and managed by BV, SE, UH, and EL. BV acts as a guarantor of the study. All authors listed above contributed to the study design and data interpretation. Writing of the first draft of the paper was done by BV and all authors were involved in the preparation and critique of the manuscript and reviewed the paper before submission.

For peer review only - http://bmjopen.bmj.com/site/about/guidelines.xhtml

Funding: This study was funded by the Medical Faculty at Uppsala University (grant number N/A), the Swedish National Stroke Association (Strokeförbundet) (grant number N/A), and the Geriatric Funding, Sweden (grant number N/A).

Patient consent for publication: Not applicable.

Competing interests: The author(s) report that there are no competing interests to declare.

Ethics approval: This study involves human participants and was approved by the regional Ethical Review Board of Uppsala University Hospital, Sweden: Dnr: 2015/550.

Data availability statement: The data set associated with this work is available from the corresponding author upon reasonable request.

ORCiD iDs

BV: http://orcid.org/0000-0002-1508-1435

SE: http://orcid.org/0000-0003-3572-8918

UH: http://orcid.org/0000-0002-0764-5878

EL: http://orcid.org/0000-0002-5313-9052

Figure: None.

For peer review only

For peer review only - http://bmjopen.bmj.com/site/about/guidelines.xhtml

References

1. Virani SS, Alonso A, Aparicio HJ, Benjamin EJ, Bittencourt MS, Callaway CW, et al. Heart Disease and Stroke Statistics-2021 Update: A Report From the American Heart Association. Circulation. 2021;143(8):e254-e743.

2. RIKSstroke. Stroke och TIA. Riksstrokes årsrapport från 2022 års data. Umeå; Riksstroke 2023. available from: htpp:// Riksstrokes-Arsrapport-2022.pdf. (cited 2 February, 2024).

3. O'Donnell MJ, Chin SL, Rangarajan S, Xavier D, Liu L, Zhang H, et al. Global and regional effects of potentially modifiable risk factors associated with acute stroke in 32 countries (INTERSTROKE): a case-control study. Lancet. 2016;388(10046):761-75.

4. McKee M, Leon DA, Tomkins S, Shkolnikov VM, Andreev E. INTERHEART. Lancet. 2005;365(9454):117-8; author reply 8.

5. Tsao CW, Aday AW, Almarzooq ZI, Anderson CAM, Arora P, Avery CL, et al. Heart Disease and Stroke Statistics-2023 Update: A Report From the American Heart Association. Circulation. 2023;147(8):e93-e621.

6. English C, Manns PJ, Tucak C, Bernhardt J. Physical activity and sedentary behaviors in people with stroke living in the community: a systematic review. Phys Ther. 2014;94(2):185-96.

7. Tudor-Locke C, Bassett DR. How many steps/day are enough? Preliminary pedometer indices for public health. Sports Med. 2004;34(1):1-8.

8. Saunders DH, Sanderson M, Hayes S, Kilrane M, Greig CA, Brazzelli M, et al. Physical fitness training for stroke patients. Cochrane Database Syst Rev. 2016;3:CD003316.

9. Field N, N G, T SS. Physical activity after stroke: A systematic review and meta-analysis. ISRN Stroke. 2013.;1.

10. Fulk GD, He Y, Boyne P, Dunning K. Predicting Home and Community Walking Activity Poststroke. Stroke. 2017;48(2):406-11.

11. Saunders DH, Sanderson M, Hayes S, Johnson L, Kramer S, Carter DD, et al. Physical fitness training for stroke patients. Cochrane Database Syst Rev. 2020;3(3):CD003316.

12. van de Port I, Punt M, Meijer JW. Walking activity and its determinants in free-living ambulatory people in a chronic phase after stroke: a cross-sectional study. Disabil Rehabil. 2020;42(5):636-41.

13. Vahlberg B, Lundström E, Eriksson S, Holmbäck U, Cederholm T. Effects on walking performance and lower body strength by short message service guided training after stroke or transient ischemic attack (The STROKEWALK Study): a randomized controlled trial. Clin Rehabil. 2021;35(2):276-87.

14. Cuschieri S. The STROBE guidelines. Saudi J Anaesth. 2019;13(Suppl 1):S31-S4.

15. Brooks D, Solway S, Gibbons W. ATS statement on six-minute walk test. Am J Respir Crit Care Med. 2003;167(9):1287.

16. Banks JL, Marotta CA. Outcomes validity and reliability of the modified Rankin scale: implications for stroke clinical trials: a literature review and synthesis. Stroke. 2007;38(3):1091-6.

17. Nasreddine ZS, Phillips NA, Bédirian V, Charbonneau S, Whitehead V, Collin I, et al. The Montreal Cognitive Assessment, MoCA: a brief screening tool for mild cognitive impairment. J Am Geriatr Soc. 2005;53(4):695-9.

18. Goldstein LB, Samsa GP, Matchar DB, Horner RD. Charlson Index comorbidity adjustment for ischemic stroke outcome studies. Stroke. 2004;35(8):1941-5.

For peer review only - http://bmjopen.bmj.com/site/about/guidelines.xhtml

19. Guralnik JM, Simonsick EM, Ferrucci L, Glynn RJ, Berkman LF, Blazer DG, et al. A short physical performance battery assessing lower extremity function: association with self-reported disability and prediction of mortality and nursing home admission. J Gerontol. 1994;49(2):M85-94.
20. Guralnik JM, Ferrucci L, Simonsick EM, Salive ME, Wallace RB. Lower-extremity function in persons over the age of 70 years as a predictor of subsequent disability. N Engl J Med. 1995;332(9):556-61.
21. Studenski S, Perera S, Patel K, Rosano C, Faulkner K, Inzitari M, et al. Gait speed and survival in older adults. JAMA. 2011;305(1):50-8.
22. Vahlberg BM, Lundström E, Eriksson S, Holmback U, Cederholm T. Potential effects on cardiometabolic risk factors and body composition by short message service (SMS)-guided training after recent minor stroke or transient ischaemic attack: post hoc analyses of the STROKEWALK randomised controlled trial. BMJ Open. 2021;11(10):e054851.
23. Cruz-Jentoft AJ, Bahat G, Bauer J, Boirie Y, Bruyère O, Cederholm T, et al. Sarcopenia: revised European consensus on definition and diagnosis. Age Ageing. 2019;48(4):601.
24. Raisi A, Zerbini V, Myers J, Masotti S, Piva T, Lordi R, et al. Moderate walking speed and survival association across 23-years follow-up in female patients with cardiovascular disease. Int J Cardiol. 2023;371:371-6.
25. Fini NA, Holland AE, Keating J, Simek J, Bernhardt J. How Physically Active Are People Following Stroke? Systematic Review and Quantitative Synthesis. Phys Ther. 2017;97(7):707-17.
26. Rojer AGM, Ramsey KA, Trappenburg MC, van Rijssen NM, Otten RHJ, Heymans MW, et al. Instrumented measures of sedentary behaviour and physical activity are associated with mortality in community-dwelling older adults: A systematic review, meta-analysis and meta-regression analysis. Ageing Res Rev. 2020;61:101061.
27. Kennedy C, Bernhardt J, Churilov L, Collier JM, Ellery F, Rethnam V, et al. Factors associated with time to independent walking recovery post-stroke. J Neurol Neurosurg Psychiatry. 2021;92(7):702-8.
28. Del Pozo Cruz B, Ahmadi MN, Lee IM, Stamatakis E. Prospective Associations of Daily Step Counts and Intensity With Cancer and Cardiovascular Disease Incidence and Mortality and All-Cause Mortality. JAMA Intern Med. 2022;182(11):1139-48.

For peer review only

For peer review only - http://bmjopen.bmj.com/site/about/guidelines.xhtml

Table 1. Baseline characteristics of all patients in the study with ≥34 meters increase in the six-minute walking test at three months versus patients with < 34 meters increase in the six-minute walking test.

	**Study population**	**Changes in the 6-minute walking test**			
	**Baseline**	**≥ 34 m (n=38)**	**<34 m (n=41)**	**P-value**	**Missing values,** n (%)
Age, mean (SD) [b]	63.9 (10.4)	61.9 (9.1)	65.7 (11.2)	0.01	0
Female, n (%) [c]	29 (36.7)	11 (28.9)	18 (43.9)	0.17	0
SMS group, n (%) [c]	40 (50.6)	26 (68.4)	14 (34.1)	0.002	0
Control group, n (%) [c]	39 (49.4)	12 (31.6)	27 (65.9)		
modified Rankin Scale, 0-2 [c]				0.47	0
0	11 (13.9)	6 (15.8)	5 (12.2)		
1	53 (67.1)	23 (60.5)	30 (73.2)		
2	15 (19.0)	9 (23.7)	6 (14.6)		
Diagnosis, n (%) [c]				0.70	0
Cerebral infarction	57 (83.5)	28 (73.7)	29 (70.7)		
Intracerebral hemorrhage	9 (11.4)	5 (13.2)	4 (9.8)		
TIA	13 (16.5)	5 (13.2)	8 (19.5)		

For peer review only

For peer review only - http://bmjopen.bmj.com/site/about/guidelines.xhtml

Charlson Comorbidity Index, n (%) [c]					0
No comorbidity	47 (59.5)	25 (65.8)	22 (53.7)	0.27	
≥1	32 (40.5)	13 (34.2)	19 (46.3)		
BMI, mean (SD) [a]	27.51 (4.5)	26.56 (3.77)	28.39 (5.03)	0.07	0
Diabetes mellitus-2, yes n (%) [c]	12 (15.2)	5 (13.2)	7 (17.1)	0.63	0
University studies, yes n (%) [c]	40 (50.6)	16 (42.1)	24 (58.5)	0.14	0
Non-smoking, n (%) [c]	71 (89.9)	33 (86.8)	38 (92.7)	0.39	0
Step counts (SMS group), mean (SD) [a]	6335 (2747)	6612 (2741)	5757 (2786)	0.38	3
SGPALS, n (%) [c]				0.47	0
Sedentary	11 (13.9)	6 (15.8)	5 (12.2)		
Light Physical Activity	53 (67.1)	23 (60.5)	30 (73.2)		
Moderate/High Physical Activity	15 (19.0)	9 (23.7)	6 (14.6)		
P-HDL cholesterol, (mmol/l), mean (SD) [b]	1.32 (0.39)	1.35 (0.45)	1.28 (0.34)	0.44	3 (3.8)
P-LDL cholesterol, (mmol/l), mean (SD) [b]	3.16 (1.09)	3.18 (1.16)	3.13 (1.03)	0.86	3 (3.8)
P-Cholesterol, (mmol/l), mean (SD) [b]	5.13 (1.20)	5.13 (1.30)	5.13 (1.12)	0.86	3 (3.8)
P-Triglycerides, (mmol/l), mean (SD) [b]	1.35 (0.63)	1.20 (0.46)	1.49 (0.73)	0.03	3 (3.8)
P-HbA1C, mmol/mol), mean (SD) [b]	38.0 (7.54)	37.74 (8.33)	38.26 (6.77)	0.47	3 (3.8)
P-C reactive protein (mg/L), mean (SD) [b]	4.92 (19.40)	6.80 (27.80)	3.20 (3.95)	0.20	6 (7.6)
P-Creatinine (mmol/L), mean (SD) [a]	85.12 (25.53)	78.97 (17.83)	90.80 (30.14)	0.042	2 (2.6)
SBP, (mm HG), mean (SD) [a]	130.63(16.56)	129.83 (16.17)	131.36 (17.09)	0.69	4 (5)
DBP, (mm HG), mean (SD) [b]	76.08 (11.0)	79.17 (11.24)	73.23 (10.09)	0.02	4 (5)

Abbreviations: BMI, body mass index, CRP, C-reactive protein; DBP, diastolic blood pressure; HbA1C, glycated hemoglobin; HDL, high-density lipoprotein; LDL, low-density lipoprotein; SGPALS, Saltin Grimby Physical Activity Scale; SMS, short message service group; SBP, systolic blood pressure.

For peer review only - http://bmjopen.bmj.com/site/about/guidelines.xhtml

For peer review only

[a] The Student´s t-test was used for continuous, normally distributed variables.

[b] The Mann Whitney-U test was applied for ordinal or non-normally distributed variables.

[c] The Chi-square test was used for categorical variables.

For peer review only

For peer review only - http://bmjopen.bmj.com/site/about/guidelines.xhtml

502 **Table 2.** Correlation of the variables used in the regression models and changes in walking
503 performance.

	**Change 6-minute walking test, *r***	**P-value**
Age (years) [b]	-0.39	<0.001
Sex (female) [b]	-0.13	0.26
CCI (≥1 comorbidity) [b]	-0.20	0.08
BMI, (kg/m^2) [a]	-0.23	0.046
6-minute walking test, baseline (meters) [a]	-0.38	<0.001
Chair-stand test, (seconds) [a]	0.10	0.39
Montreal Cognitive Assessment scale, (≥26 points), [b]	0.014	0.91
Saltin Grimby Physical Activity Level Scale [b] (Sedentary, light physical activity, moderate/high physical activity)	0.078	0.49

504 Abbreviations: BMI, Body Mass Index; CCI, Charlson Comorbidity Index

505 [a] Correlation strength was calculated using Spearman´s rho for ordinal and non-normally
506 distributed variables

507 [b] Correlation strength was calculated using Pearson correlation for continuous and normally
508 distributed variables

509

510

For peer review only - http://bmjopen.bmj.com/site/about/guidelines.xhtml

For peer review only

Table 3. Univariate and multivariate regression analyses with changes in walking performance (6-minute walking test) as the dependent variable and age, sex, comorbidity, baseline BMI-, and baseline 6-minute walk test as independent variables in individuals after stroke and TIA. An intention-to-treat analysis was performed with drop-outs included (n=8).

	**Univariate analysis**				**Multivariate analysis**		
	**Beta standardized**	**Adjusted R square**	**95% CI**	**P**	**Beta standardized**	**95% CI**	**P**
Age, yrs	-0.20	0.029	-3.1 to 0.1	0.073	-0.33	-3.8 to -1.0	<0.001
Sex, female	-0.12	0.002	-54.4 to 16.4	0.29	-0.24	-66.9 to -8.0	0.014
CCI, ≥1 comorbidity	-0.19	0.024	-63.8 to 4.9	0.09	-0.16	-55.5 to 5.4	0.11
BMI, kg/m^2	-0.23	0.038	-7.5 to -0.07	0.046	-0.29	-8.1 to -1.6	0.004
6-minute walk test, meters	-0.38	0.134	-0.4 to -0.1	<0.001	-0.55	-0.5 to -0.3	<0.001

Abbreviations: BMI, Body Mass Index; CCI, Charlson Comorbidity Index

The adjusted R square for the multivariate analysis is 0.36.

For peer review only

Table 4. Sensitivity analysis: Univariate and multivariate regression analyses with changes in walking performance (6-minute walking test) as the dependent variable and age, sex, comorbidity, baseline BMI-, and baseline 6-minute walk test as independent variables in individuals after stroke and TIA. In the sensitivity analysis, individuals with missing data at three months were not analyzed (n=8)

	**Univariate analysis**				**Multivariate analysis**		
	**Beta standardized**	**Adjusted R square**	**95% CI**	**P**	**Beta standardized**	**95% CI**	**P**
Age, yrs	-0.25	0.05	-3.9 to -0.2	0.03	-0.33	-4.3 to -1.1	0.002
Sex, female	-0.11	-0.002	-57.5 to 20.8	0.35	-0.24	-72.7 to -7.1	0.018
CCI, ≥1 comorbidity	-0.18	0.018	-66.6 to 9.7	0.14	-0.22	-67.4 to -2.6	0.035
BMI, kg/m^2	-0.21	0.031	-8.1 to 0.43	0.08			
6-minute walk test, meters	-0.42	0.16	-0.47 to -0.15	<0.001	-0.55	-0.56 to -0.26	<0.001

Abbreviations: BMI, Body Mass Index; CCI, Charlson Comorbidity Index

The adjusted R square for the multivariate analysis is 0.34.

For peer review only

Print Form

CONSORT CHECKLIST

Table. CONSORT 2010 Checklist of Information to Include When Reporting a Randomized Trial[a]

Section and Topic	Item No.	Checklist Item	Reported on Page No.
**Title and abstract**			
	1a	Identification as a randomized trial in the title	title pag
	1b	Structured summary of trial design, methods, results, and conclusions (for specific guidance see CONSORT for abstracts)	abstract
**Introduction**			
Background and objectives	2a	Scientific background and explanation of rationale	p. 1-2
	2b	Specific objectives or hypotheses	p. 1-2
**Methods**			
Trial design	3a	Description of trial design (such as parallel, factorial) including allocation ratio	p. 2
	3b	Important changes to methods after trial commencement (such as eligibility criteria), with reasons	ref 13
Participants	4a	Eligibility criteria for participants	p. 2
	4b	Settings and locations where the data were collected	p. 2
Interventions	5	The interventions for each group with sufficient details to allow replication, including how and when they were actually administered	p. 4-5
Outcomes	6a	Completely defined prespecified primary and secondary outcome measures, including how and when they were assessed	p. 3-4
	6b	Any changes to trial outcomes after the trial commenced, with reasons	N/A
Sample size	7a	How sample size was determined	p. 5
	7b	When applicable, explanation of any interim analyses and stopping guidelines	N/A
Randomization			
Sequence generation	8a	Method used to generate the random allocation sequence	ref 13
	8b	Type of randomization; details of any restriction (such as blocking and block size)	ref 13
Allocation concealment mechanism	9	Mechanism used to implement the random allocation sequence (such as sequentially numbered containers), describing any steps taken to conceal the sequence until interventions were assigned	ref 13
Implementation	10	Who generated the random allocation sequence, who enrolled participants, and who assigned participants to interventions	ref 13
Blinding	11a	If done, who was blinded after assignment to interventions (for example, participants, care providers, those assessing outcomes) and how	ref 13
	11b	If relevant, description of the similarity of interventions	N/A
Statistical methods	12a	Statistical methods used to compare groups for primary and secondary outcomes	p. 5 and
	12b	Methods for additional analyses, such as subgroup analyses and adjusted analyses	p. 6
**Results**			
Participant flow (a diagram is strongly recommended)	13a	For each group, the numbers of participants who were randomly assigned, received intended treatment, and were analyzed for the primary outcome	ref 13
	13b	For each group, losses and exclusions after randomization, together with reasons	ref 13
Recruitment	14a	Dates defining the periods of recruitment and follow-up	ref 13, P
	14b	Why the trial ended or was stopped	N/A
Baseline data	15	A table showing baseline demographic and clinical characteristics for each group	Table 1
Numbers analyzed	16	For each group, number of participants (denominator) included in each analysis and whether the analysis was by original assigned groups	ref 13
Outcomes and estimation	17a	For each primary and secondary outcome, results for each group, and the estimated effect size and its precision (such as 95% confidence interval)	ref 13
	17b	For binary outcomes, presentation of both absolute and relative effect sizes is recommended	N/A
Ancillary analyses	18	Results of any other analyses performed, including subgroup analyses and adjusted analyses, distinguishing prespecified from exploratory	T 4
Harms	19	All important harms or unintended effects in each group (for specific guidance see CONSORT for harms)	N/A
**Comment**			
Limitations	20	Trial limitations, addressing sources of potential bias, imprecision, and, if relevant, multiplicity of analyses	below al
Generalizability	21	Generalizability (external validity, applicability) of the trial findings	p.11
Interpretation	22	Interpretation consistent with results, balancing benefits and harms, and considering other relevant evidence	ref 13
**Other information**			
Registration	23	Registration number and name of trial registry	abstract
Protocol	24	Where the full trial protocol can be accessed, if available	pilot stu
Funding	25	Sources of funding and other support (such as supply of drugs), role of funders	p. 12

[a] We strongly recommend reading this statement in conjunction with the CONSORT 2010 Explanation and Elaboration for important clarifications on all the items. If relevant, we also recommend reading CONSORT extensions for cluster randomized trials, noninferiority and equivalence trials, nonpharmacological treatments, herbal interventions, and pragmatic trials. Additional extensions are forthcoming: for those and for up-to-date references relevant to this checklist, see http://www.consort-statement.org.

©2010 American Medical Association. All rights reserved.

For peer review only

For peer review only - http://bmjopen.bmj.com/site/about/guidelines.xhtml

STROBE Statement—checklist of items that should be included in reports of observational studies

	Item No	Recommendation
**Title and abstract**	1	(*a*) Indicate the study's design with a commonly used term in the title or the abstract
		(*b*) Provide in the abstract an informative and balanced summary of what was done and what was found
**Introduction**		
Background/rationale	2	Explain the scientific background and rationale for the investigation being reported
Objectives	3	State specific objectives, including any prespecified hypotheses
**Methods**		
Study design	4	Present key elements of study design early in the paper
Setting	5	Describe the setting, locations, and relevant dates, including periods of recruitment, exposure, follow-up, and data collection
Participants	6	(*a*) *Cohort study*—Give the eligibility criteria, and the sources and methods of selection of participants. Describe methods of follow-up<br>*Case-control study*—Give the eligibility criteria, and the sources and methods of case ascertainment and control selection. Give the rationale for the choice of cases and controls<br>*Cross-sectional study*—Give the eligibility criteria, and the sources and methods of selection of participants
		(*b*) *Cohort study*—For matched studies, give matching criteria and number of exposed and unexposed<br>*Case-control study*—For matched studies, give matching criteria and the number of controls per case
Variables	7	Clearly define all outcomes, exposures, predictors, potential confounders, and effect modifiers. Give diagnostic criteria, if applicable
Data sources/ measurement	8*	For each variable of interest, give sources of data and details of methods of assessment (measurement). Describe comparability of assessment methods if there is more than one group
Bias	9	Describe any efforts to address potential sources of bias
Study size	10	Explain how the study size was arrived at
Quantitative variables	11	Explain how quantitative variables were handled in the analyses. If applicable, describe which groupings were chosen and why
Statistical methods	12	(*a*) Describe all statistical methods, including those used to control for confounding
		(*b*) Describe any methods used to examine subgroups and interactions
		(*c*) Explain how missing data were addressed
		(*d*) *Cohort study*—If applicable, explain how loss to follow-up was addressed<br>*Case-control study*—If applicable, explain how matching of cases and controls was addressed<br>*Cross-sectional study*—If applicable, describe analytical methods taking account of sampling strategy
		(*e*) Describe any sensitivity analyses

Continued on next page

For peer review only

For peer review only - http://bmjopen.bmj.com/site/about/guidelines.xhtml

**Results**		
Participants	13*	(a) Report numbers of individuals at each stage of study—eg numbers potentially eligible, examined for eligibility, confirmed eligible, included in the study, completing follow-up, and analysed
		(b) Give reasons for non-participation at each stage
		(c) Consider use of a flow diagram
Descriptive data	14*	(a) Give characteristics of study participants (eg demographic, clinical, social) and information on exposures and potential confounders
		(b) Indicate number of participants with missing data for each variable of interest
		(c) *Cohort study*—Summarise follow-up time (eg, average and total amount)
Outcome data	15*	*Cohort study*—Report numbers of outcome events or summary measures over time
		Case-control study—Report numbers in each exposure category, or summary measures of exposure
		Cross-sectional study—Report numbers of outcome events or summary measures
Main results	16	(*a*) Give unadjusted estimates and, if applicable, confounder-adjusted estimates and their precision (eg, 95% confidence interval). Make clear which confounders were adjusted for and why they were included
		(*b*) Report category boundaries when continuous variables were categorized
		(*c*) If relevant, consider translating estimates of relative risk into absolute risk for a meaningful time period
Other analyses	17	Report other analyses done—eg analyses of subgroups and interactions, and sensitivity analyses
**Discussion**		
Key results	18	Summarise key results with reference to study objectives
Limitations	19	Discuss limitations of the study, taking into account sources of potential bias or imprecision. Discuss both direction and magnitude of any potential bias
Interpretation	20	Give a cautious overall interpretation of results considering objectives, limitations, multiplicity of analyses, results from similar studies, and other relevant evidence
Generalisability	21	Discuss the generalisability (external validity) of the study results
**Other information**		
Funding	22	Give the source of funding and the role of the funders for the present study and, if applicable, for the original study on which the present article is based

*Give information separately for cases and controls in case-control studies and, if applicable, for exposed and unexposed groups in cohort and cross-sectional studies.

Note: An Explanation and Elaboration article discusses each checklist item and gives methodological background and published examples of transparent reporting. The STROBE checklist is best used in conjunction with this article (freely available on the Web sites of PLoS Medicine at http://www.plosmedicine.org/, Annals of Internal Medicine at http://www.annals.org/, and Epidemiology at http://www.epidem.com/). Information on the STROBE Initiative is available at www.strobe-statement.org.

For peer review only

For peer review only - http://bmjopen.bmj.com/site/about/guidelines.xhtml
